# A study of genetic heterogeneity in autism spectrum disorders based on plasma proteomic and metabolomic analysis: multiomics study of autism heterogeneity

**DOI:** 10.1002/mco2.380

**Published:** 2023-09-24

**Authors:** Xiaoxiao Tang, Chengyun Feng, Yuxi Zhao, Huajie Zhang, Yan Gao, Xueshan Cao, Qi Hong, Jing Lin, Hongbin Zhuang, Yuying Feng, Hanghang Wang, Liming Shen

**Affiliations:** ^1^ College of Life Science and Oceanography Shenzhen University Shenzhen P. R. China; ^2^ Maternal and Child Health Hospital of Baoan Shenzhen P. R. China; ^3^ Shenzhen‐Hong Kong Institute of Brain Science‐Shenzhen Fundamental Research Institutions Shenzhen P. R. China; ^4^ Shenzhen Key Laboratory of Marine Biotechnology and Ecology Shenzhen P. R. China

**Keywords:** autism spectrum disorder, biomarker, heterogeneity, metabolomics, plasma, proteomics

## Abstract

Genetic heterogeneity poses a challenge to research and clinical translation of autism spectrum disorder (ASD). In this study, we conducted a plasma proteomic and metabolomic study of children with ASD with and without risk genes (*de novo* mutation) and controls to explore the impact of genetic heterogeneity on the search for biomarkers for ASD. In terms of the proteomic and metabolomic profiles, the groups of children with ASD carrying and those not carrying *de novo* mutation tended to cluster and overlap, and integrating them yielded differentially expressed proteins and differential metabolites that effectively distinguished ASD from controls. The mechanisms associated with them focus on several common and previously reported mechanisms. Proteomics results highlight the role of complement, inflammation and immunity, and cell adhesion. The main pathways of metabolic perturbations include amino acid, vitamin, glycerophospholipid, tryptophan, and glutamates metabolic pathways and solute carriers‐related pathways. Integrating the two omics analyses revealed that L‐glutamic acid and malate dehydrogenase may play key roles in the pathogenesis of ASD. These results suggest that children with ASD may have important underlying common mechanisms. They are not only potential therapeutic targets for ASD but also important contributors to the study of biomarkers for the disease.

## Graphical abstract

Based on genetic testing, plasma proteomics and metabolomics were studied in children with autism spectrum disorder (ASD) and controls. The omics characteristics of children with ASD carrying and not carrying risk genes were similar. The differential proteins or metabolites obtained between ASD and controls were clustered in several disease‐related mechanisms, which have the potential to be biomarkers of ASD.

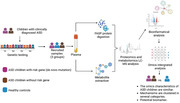

## 1 INTRODUCTION

Autism spectrum disorder (ASD) is a complex neurodevelopmental disorder characterized by repetitive behavior, limited interests, and difficulty communicating and reciprocating with others.^1^ Its prevalence has gradually increased in recent years, with a 4:1 ratio of male to female.^2^ The exact pathogenesis of ASD is not yet well known.^3^ Twin studies suggest that genes play an important role in the pathogenesis of ASD.^4^ Environmental factors can also contribute to ASD. It also may be the result of interaction between genes and environmental factors, such as epigenetic modifications of genes caused by environmental factors.^5^

There are currently no unbiased biomarkers available for the diagnosis of ASD. Early intervention has been reported to lead to rapid improvement in children with ASD.^6^, ^7^ Therefore, it is important to identify common biomarkers or objective indicators of ASD. Blood is rich in substances that can be easily sampled and are suitable candidates when screening for disease, and there have been some studies of blood protein‐ or metabolite‐based diagnostic markers for ASD.^8^, ^9^, ^10^ However, due to the heterogeneity of ASD, it is challenging to find diagnostic biomarkers of ASD. The heterogeneity of ASD is characterized by heterogeneity in the etiology, phenotype, and outcome.^11^ Genetic variation, gender, and co‐morbidity contribute to the heterogeneity of ASD. Of these, genetic variation is thought to be the principal factor,^12^ which includes single gene disorders, copy number variants (CNVs), inherited and *de novo* rare variants, and common sequence variants,^8^, ^11^ whose differences lead to genetic heterogeneity. For example, there may be thousands of genes involved in ASD^8^; however, each gene alone accounts for less than 1% of cases.^5^

Genetic heterogeneity makes the study of plasma biomarkers of ASD complex and difficult. It may be inconclusive to combine data from children with different genetic etiologies. However, common features or disease mechanisms may still be found among these children. Finding the “common ground” among children with ASD has become the breakthrough point, focus, and hotspot of current research. Efforts have been made to identify biomarkers and explore the possibility of defining ASD subgroups by biological characteristics.^8^, ^9^, ^11^, ^13^, ^14^, ^15^, ^16^ Several genes associated with ASD are found in common biological processes (BPs) and pathways, such as brain development, regulation of gene expression (e.g., chromatin modification and alternative splicing), neuronal and synaptic formation and function, and synaptic neuronal communication, implying that they may be important to the pathogenesis of ASD.^14^, ^15^, ^16^, ^17^, ^18^ The spatiotemporal expression analysis of autism risk genes showed that they have a trend of co‐expression in early embryonic brain development.^14^ Besides, genes, CNVs, and common variants could be clustered into discrete groups.^14^ In addition, there have been some reports that support the existence of behaviorally and genetically distinct subgroups.^19^, ^20^ The association analysis between the dynamic characteristics of brain functional network and gene expression profiles revealed the consistent changes in brain network dynamics in ASD and the transcriptomic characteristics related to these changes.^13^ Based on these shared molecular mechanisms, biomarkers for searching and diagnosing diseases as well as targeted therapeutics can be developed.

Proteomics and metabolomics are high‐throughput detection technologies. On the basis of gene detection, the research on the correlation between gene heterogeneity and omics characteristics is very conducive to finding common ground for children with ASD, exploring disease mechanisms and looking for diagnostic markers. Using a single‐cell RNA‐sequencing analysis and proteomic approach, a recent study investigated the induced pluripotent stem cell‐derived “brain‐like organs” from children with three different ASD risk genes,^21^ which showed that various ASD risk genes are focused on the phenotype of neuronal developmental asynchrony but mostly diverge at the level of molecular targets.^21^ Very recently, we carried out proteomic and metabolomic studies in the plasma of ASD children with five different risk genes (*de novo* mutations), as well as proteomics studies of peripheral blood mononuclear cells.^22^ The results show that children with ASD were heterogeneous at the genetic level, but differentially expressed proteins (DEPs) and differential metabolites in plasma could still be used to differentiate cases from controls.^22^ These two studies provide a paradigm for the study of genetic heterogeneity.

However, the group of children with ASD but without the risk gene was lacking in our previous study.^22^ To more comprehensively explore the association of plasma proteomic and metabolomic features with genetic heterogeneity, and the impact of genetic heterogeneity on the search for blood biomarkers, in the present study, we added this group. A total of three groups were generated: ASD children with risk gene (ASD_M group), ASD children without risk gene (ASD_nM group), and healthy control group (CTR group). Plasma proteomics and metabolomics studies were carried out by applying the sequential window acquisition of all theoretical fragment ions (SWATH) mass spectrometry (MS) technique and high‐performance liquid chromatography‐MS (HPLC‐MS), respectively. More total proteins and DEPs were identified, compared to the previous study.^22^ The levels of total metabolites and differential metabolites were positively correlated between the ASD groups carrying the risk gene and those not carrying the risk gene, which better indicates the similarity between these two groups. DEPs and differential metabolites were focused on several previously reported mechanisms suggesting the existence of common mechanisms, and they may become diagnostic markers for ASD.

## 2 RESULTS

### 2.1 The clinical characteristics of children with ASD matched those of controls

**FIGURE 1:**
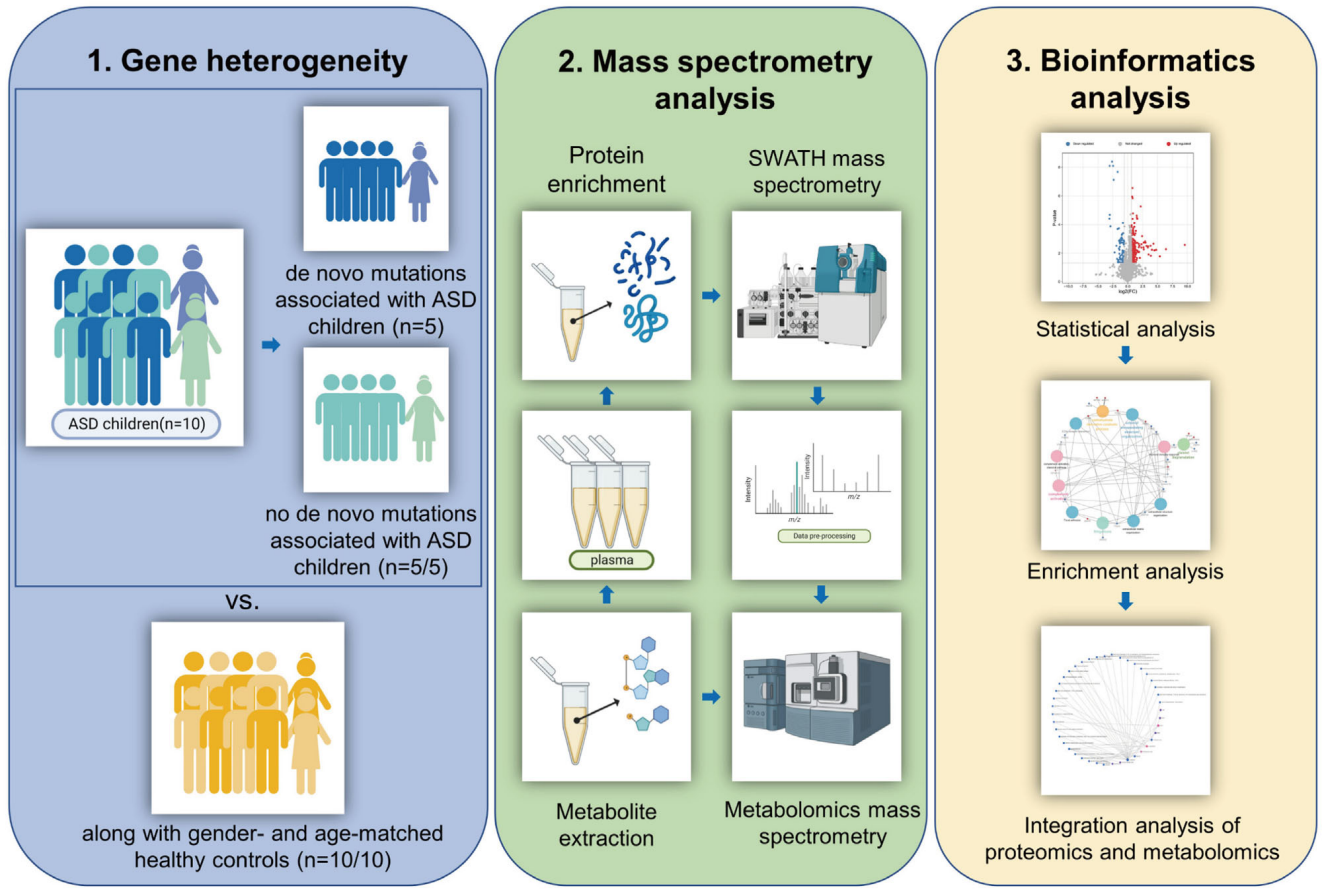
An overview of the workflow used in this study. On the basis of genetic detection, plasma proteomic and metabolomic analyses were performed in autism spectrum disorder (ASD) children with risk genes (*de novo* mutation) and age‐ and sex‐matched ASD children with no risk genes and their controls, by using the sequence window to obtain all theoretical fragment ions (SWATH) technique and high‐performance liquid chromatography‐mass spectrometry (HPLC‐MS), respectively. The mass spectrum data were then analyzed statistically and bioinformatically to obtain associations between genetic heterogeneity and omics characteristics, disease mechanisms, and potential diagnostic biomarkers.

To describe the workflow of this study, we drew a flowchart (Figure 1). On the basis of genetic testing, we performed plasma proteomics and metabolomics studies, as well as integrative omics studies in children with ASD carrying and not carrying risk genes and in controls (Figure 1). To ensure that the clinical characteristics of the study subjects were matched, their clinical characteristics were compared (Table S1). These children were diagnosed by using the Diagnostic and Statistical Manual of Mental Disorders (DSM‐V) and also assessed by the Autism Behavior Checklist (ABC) and the Childhood Autism Rating Scale (CARS).^22^, ^23^ They fit the diagnosis and characteristics of ASD. There were no significant differences in age, sex, or body mass index (BMI) between the ASD_M and ASD_nM groups, or between the ASD and control groups, suggesting that these clinical characteristics were matched. The genetic test results of the five children carrying risk genes are shown in Table S2, all of which were *de novo* gene mutations, including *DDX3X* (adenosine triphosphate dependent RNA helicase DDX3X), *GIGYF2* (GRB10‐interacting GYF protein 2), *NAA15* (N‐alpha‐acetyltransferase 15, NatA auxiliary subunit), *ASH1L* (histone‐lysine N‐methyltransferase ASH1L), and *SCN2A* (sodium channel protein type 2 subunit alpha).

### 2.2 Plasma proteomic characteristics of different groups and DEPs between the ASD group and control group were obtained

**FIGURE 2:**
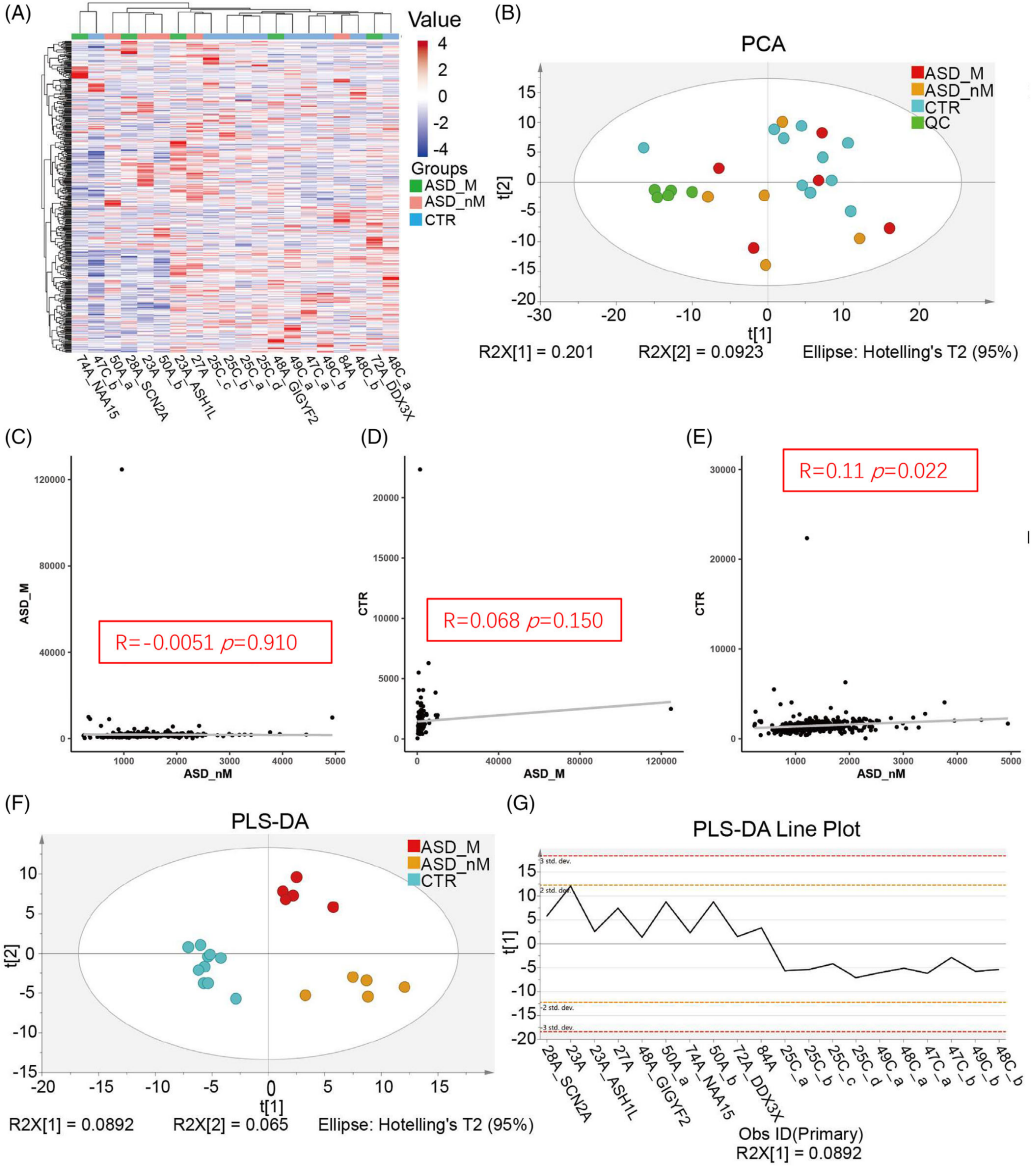
Proteomic characteristics of total proteins between different groups. (A) Cluster analysis of total proteins in ASD children with risk gene (ASD_M group), ASD children without risk gene (ASD_nM group), and control group (CTR group). The value indicates the expression of the protein, red indicates high expression, and blue indicates low expression. (B) Principal component analysis (PCA) analysis of total proteins in ASD_M group, ASD_nM group, and CTR group. (C–E) Correlation analysis of total proteins in ASD_M group, ASD_nM group, and CTR group. (F) Partial least squares discriminant analysis (PLS‐DA) analysis of total proteins in ASD_M group, ASD_nM group, and CTR group. (G) PLS‐DA scores line plot.

**FIGURE 3:**
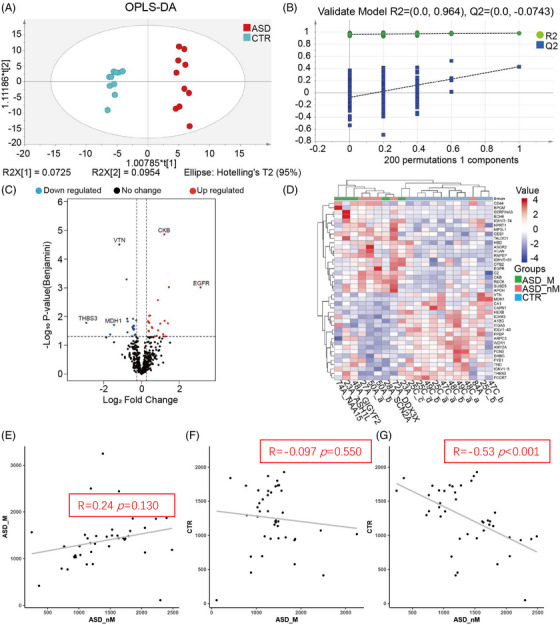
The differentially expressed proteins (DEPs) were identified between the ASD and control groups. (A) Orthogonal projections to latent structures (OPLS)‐DA analysis of total proteins in ASD and healthy controls. (B) Validation of OPLS‐DA model by 200 permutation tests. (C) Volcano plots depicted the distribution of plasma proteins between children with ASD and healthy controls. The log_2_ fold change (FC) is plotted versus the –log_10_ of the *p*‐value (Benjamini–Hochberg false discovery rate). Red dots: hits with *p* < 0.05 and mean ­log_2_FC > 0.26; blue dots: hits with *p* < 0.05 and mean |log_2_FC| < 0.26. (D) Cluster analysis of DEPs between ASD and the control groups. The value indicates the expression of the protein, red indicates high expression, and blue indicates low expression. (E–G) Correlation analysis of DEPs in ASD_M group, ASD_nM group, and CTR group.

To assess the association of proteomic characteristics with genetic heterogeneity, proteomic analysis was performed, and the classification features between groups were analyzed by multivariate statistical analysis. A total of 1747 proteins were identified, and 836 proteins were quantified. Cluster analysis of quantified proteins showed that the three groups (ASD_M, ASD_nM, and CTR) overlapped, but the control group was relatively concentrated (Figure 2A). Principal component analysis (PCA) analysis showed that quality control (QC) samples were clustered together, which means the machine error can be ignored. These three groups partially overlapped (Figure 2B). Besides, the segregation trend between the CTR group and the ASD group (including ASD_M and ASD_nM group) was stronger, while the segregation trend between ASD_M group and ASD_nM group was weaker (Figure 2B). Correlation analysis of total protein expression showed that there was a correction between ASD_nM group and CTR group (R = 0.11, *p* = 0.022), while there was no significant correlation between the other two groups (Figure 2C–E). Using the supervised analysis method partial least squares discriminant analysis (PLS‐DA), the ASD group showed a difference from the control group in the first component, but there was no difference between ASD_M group and ASD_nM group (Figure 2F). The results of the 200 permutation tests showed that this model was not overfitted (Q2 = −0.108, R2 = 0.772; Figure S1A). In the PLS‐DA scores line plot, ASD_M group and ASD_nM group were shown to have positive scores, but the CTR group had negative scores (Figure 2G). When the ASD_M and ASD_nM groups were separated by orthogonal projections to latent structures‐discriminant analysis (OPLS‐DA), the models were overfitted (Figure S1B,C). These results suggest that the two “subtypes” of ASD are not suitable for separation analysis. Therefore, we compared children with and without the risk gene as a group with healthy controls. The OPLS‐DA result showed that the ASD group and the control group could be well distinguished (Figure 3A). A low Q2 value of −0.0743 and R2 = 0.964 for the 200 permutation tests indicated that the model was not overfitted (Figure 3B).

A total of 41 DEPs were identified from the total proteins (Figure 3C, Table S3). There were 21 up‐regulated proteins and 20 down‐regulated proteins, compared to controls. When these 41 proteins were used for cluster analysis, with the exception of one child that does not carry the risk ASD gene, they could distinguish between ASD and controls but not children with ASD risk genes and children without ASD risk genes. There was an overlap between individuals with risk and non‐risk genes (Figure 3D). As shown in Figure 3E–G, the expression of DEPs in the ASD_nM group was negatively correlated with that in the CTR group (R = −0.53, *p* < 0.001), while there was no significant correlation between the other two groups. However, the ASD_M group and the ASD_nM group showed a positive trend of correlation.

These results suggest that the total protein distribution characteristics were similar between the ASD_M and ASD_nM groups, which could be effectively distinguished from the control group when combined into one group. However, the correlation analysis of total protein expression levels between the groups was not obvious, while a positive correlation trend was observed between the ASD_M group and the ASD_nM group.

### 2.3 Bioinformatics analysis of the DEPs

**FIGURE 4:**
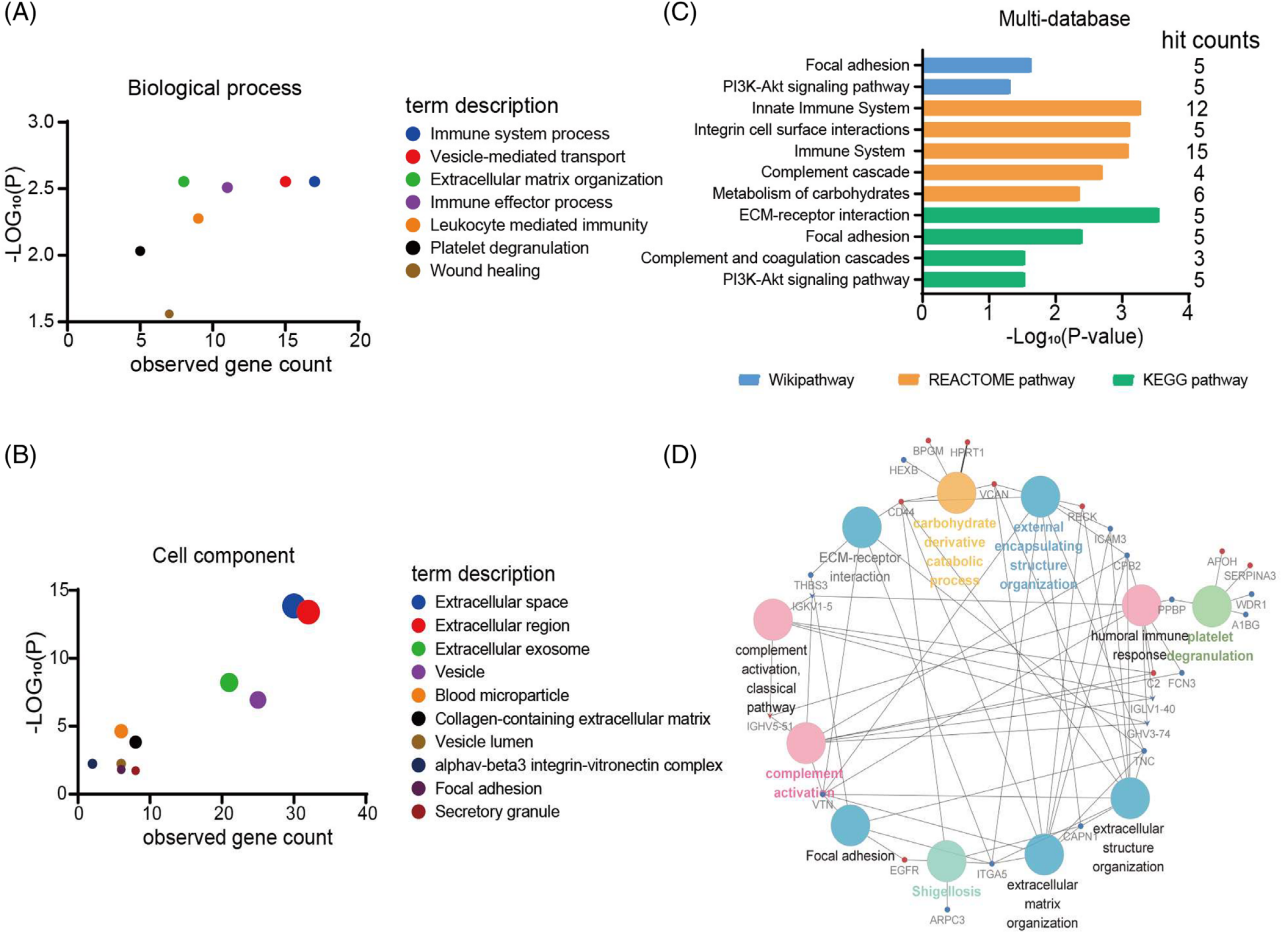
Bioinformatic analysis of the DEPs between ASD and control groups. (A) The biological processes associated with the DEPs. (B) The top 10 cellular components associated with the DEPs. (C) Important pathways associated with the DEPs in Kyoto Encyclopedia of Genes and Genomes (KEGG), REACTOME, and Wiki pathway analysis. (D) Important pathways in ClueGO analysis. The thicker the line, the stronger the interaction. Large circles represent pathways and small circles represent genes. In the small circles, red indicates up‐regulation and blue indicates down‐regulation. Immunoglobulins are shown in the shape of an arrow.

In order to access the mechanisms and BPs associated with these DEPs, we next performed a bioinformatic analysis of the DEPs. By using STRING database (version 9.1, http://string‐db.org/) analysis, as shown in Figure 4 and Table S4, the BPs linked to DEPs mainly included immune system process, vesicle‐mediated transport, extracellular matrix organization, immune effector process, leukocyte mediated immunity, platelet degranulation, and wound healing (Figure 4A). The cellular components linked to DEPs mainly included extracellular space, extracellular region, extracellular exosome, vesicle, blood microparticle, collagen‐containing extracellular matrix, vesicle lumen, alphav‐beta3 integrin‐vitronectin complex, focal adhesion, and secretory granule (Figure 4B). The DEPs associated with the important Kyoto Encyclopedia of Genes and Genomes (KEGG), REACTOME, and Wiki pathways are shown in Figure 4C. These proteins were mainly related to complement and coagulation cascades, complement cascade immune system, focal adhesion, and metabolism of carbohydrates.

Functional interaction networks were constructed using the ClueGO plugin from Cytoscape software (version 3.7.1, https://cytoscape.org/), which were consistent with the analysis of BP, CC, KEGG, and Wiki pathways (Figure 4D). These proteins were mainly involved in complement activation classical pathway, complement activation, humoral immune response, extracellular matrix‐ (EMC‐) receptor interaction, external encapsulating structure organization, extracellular structure organization, platelet degranulation, extracellular matrix organization, shigellosis, and carbohydrate derivative catabolic process. Among them, CPB2, C2, FCN3, VTN, IGHV5‐51, IGHV3‐74, IGKV1‐5, and IGLV1‐40 were enriched in complement activation.

STRING database from Cytoscape software was used to identify the top 15 hub proteins from the DEPs, including ITGA5, VCAN, TNC, CD44, VTN, EGFR, CPB2, APOH, A1BG, GIG25, CKB, TALDO1, MDH1, ARPC3, and RNPEP (Figure S1D). By using STRING database analysis, they were involved in focal adhesion, extracellular matrix organization, cell adhesion, cell motility, phosphatidylinositol‐3‐kinase PI3K‐Akt signaling pathway, and metabolism of carbohydrates (Figure S1E). Bioinformatic analysis of DEPs and hub proteins showed that the gene ontology (GO) terms and signaling pathways involved were relatively concentrated.

### 2.4 Plasma metabolomics characteristics of different groups and differential metabolites between the ASD group and control group were obtained

**FIGURE 5:**
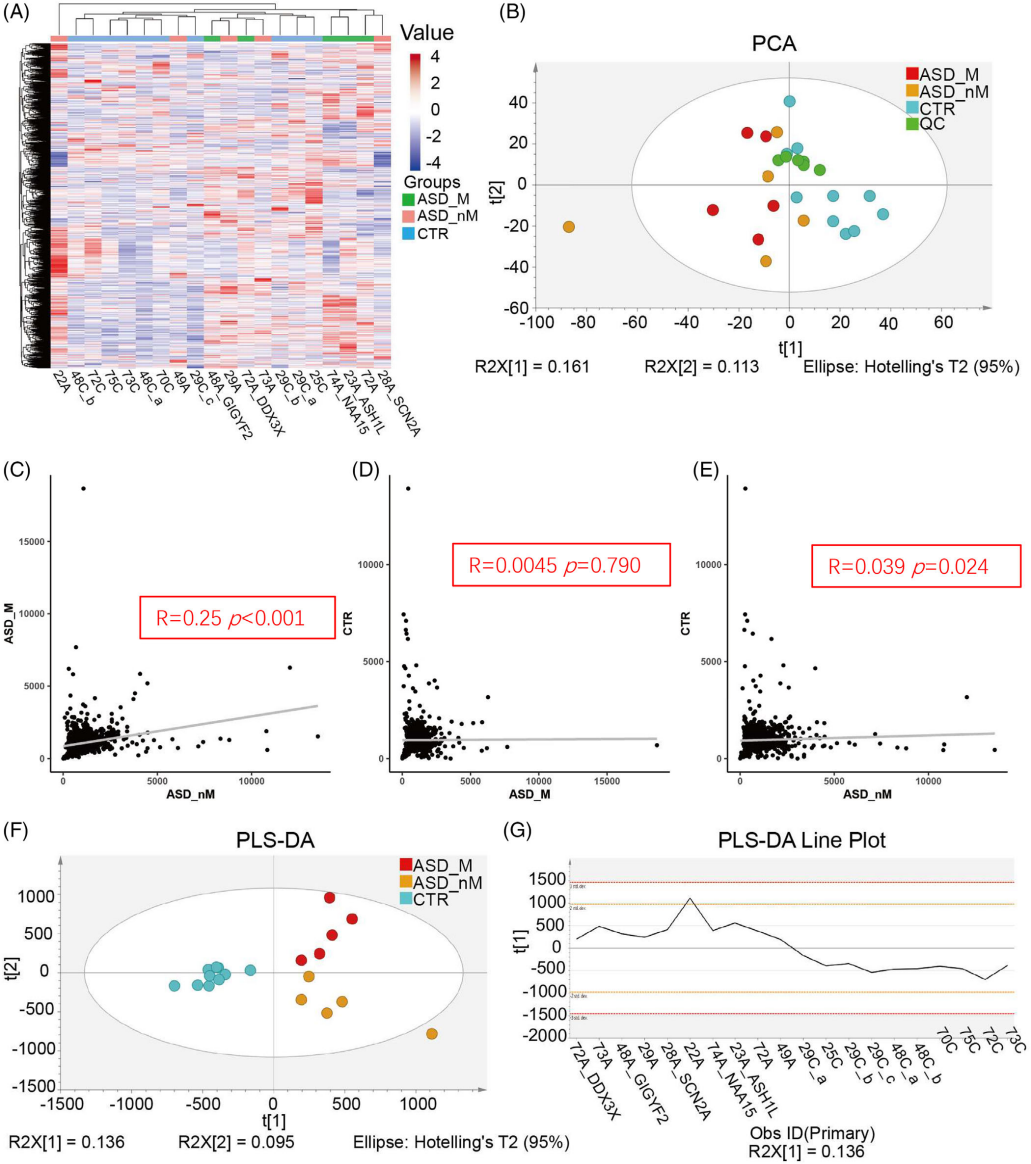
Metabolomic characteristics of total metabolites between different groups. (A) Cluster analysis of total metabolites in ASD_M group, ASD_nM group, and CTR group. The value indicates the expression of the metabolite, red indicates high expression, and blue indicates low expression. (B) PCA analysis of total metabolites in ASD_M group, ASD_nM group, and CTR group. (C–E) Correlation analysis of total metabolites in ASD_M group, ASD_nM group, and CTR group. (F) PLS‐DA analysis of total metabolites in ASD_M group, ASD_nM group, and CTR group. (G) PLS‐DA scores line plot.

**FIGURE 6:**
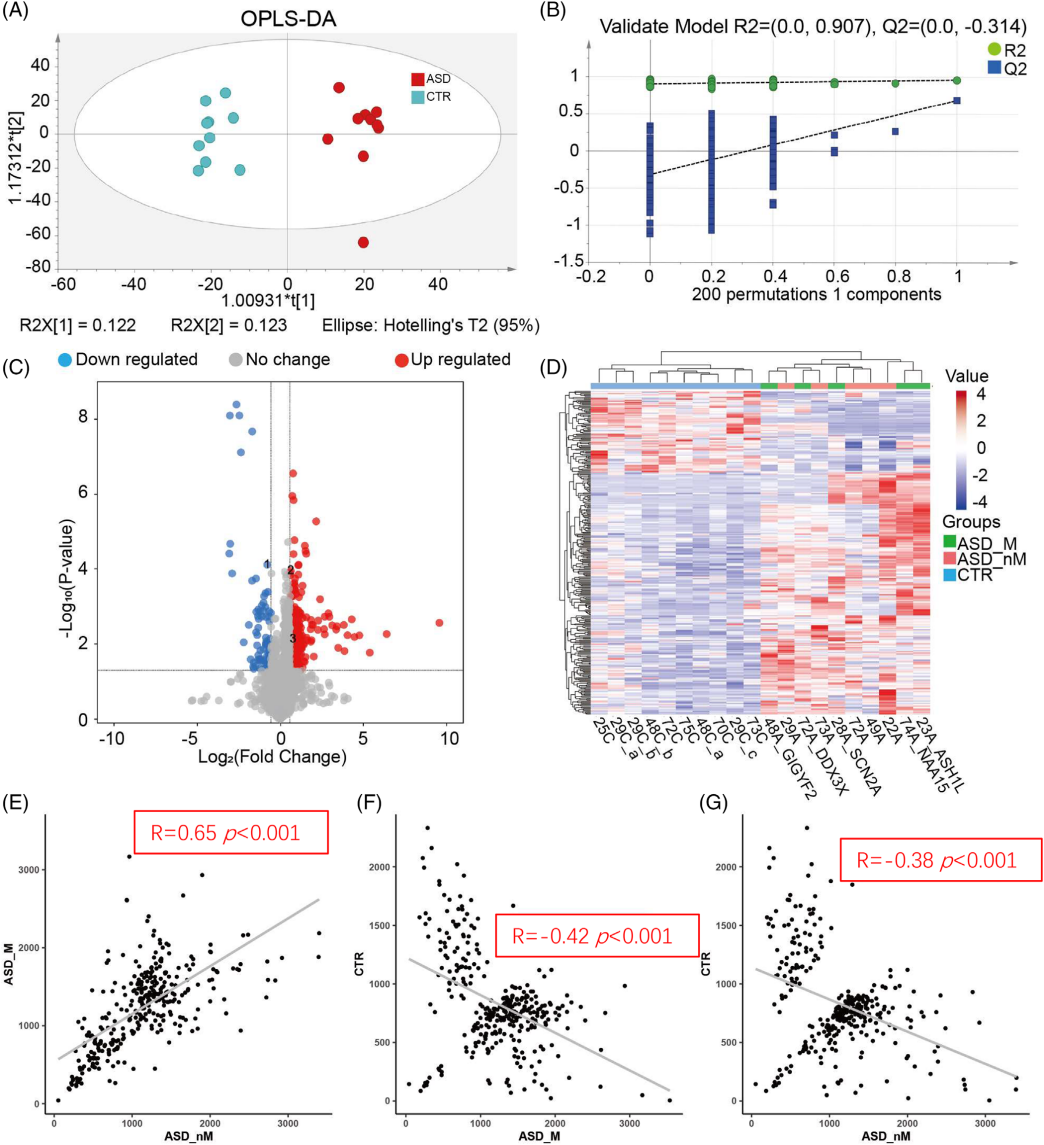
The differential metabolites were identified between the ASD and control groups. (A) OPLS‐DA analysis of total metabolites in ASD and the healthy control groups. (B) Validation of OPLS‐DA model by 200 permutation tests. (C) Volcano plots depicted the metabolite distribution of children with ASD and healthy controls. Blue dots indicate down‐regulated metabolite, and red dots indicate up‐regulated metabolite. The log_2_ FC is plotted versus the –log_10_ of the *p*‐value (Benjamini). Red dots: hits with *p* < 0.05 and mean ­log_2_FC > 0.58; blue dots: hits with *p* < 0.05 and mean |log_2_FC| < 0.58. 1 indicates L‐glutamic acid, 2 indicates D‐glucose, 3 indicates 3‐mercaptopyruvic acid. For these three metabolites, log_2_FC was about 1, that is, and the FC was about two‐fold compared to the control. (D) Cluster analysis of differential metabolites between ASD and control groups. The value indicates the expression of the metabolite, red indicates high expression, and blue indicates low expression. (E–G) Correlation analysis of differential metabolites in ASD_M group, ASD_nM group, and CTR group.

To assess the association of metabonomic characteristics with genetic heterogeneity, metabonomic analysis was performed, the classification features between groups were analyzed, and differential metabolites were identified. A total of 3399 quantitative metabolites were obtained after removing internal standards and false positive peaks and incorporating peaks from the same metabolites. The clustering heat map of the total metabolites showed that the three groups (ASD_M group, ASD_nM group, and healthy control group) overlapped; however, the control group was relatively concentrated (Figure 5A). Children with ASD were distinguished from healthy controls based on PCA analysis, while there was an overlap between ASD_M group and ASD_nM group (Figure 5B). In Figure 5C–E, the levels of total metabolites were correlated between the ASD_M and ASD_nM groups (R = 0.25, *p* < 0.001), and also between the ASD_nM and CTR groups, but with a lower R‐value (0.039). As shown in Figures 5F,G and S1F–H, the results of the PLS‐DA analysis of the three groups and the OPLS‐DA analysis of the two groups (ASD_M group and ASD_nM group) were similar to those of the proteomics study. Thus, we also combined children with and without ASD risk genes and compared them with healthy controls. OPLS‐DA analysis revealed that ASD and controls could be well differentiated (Figure 6A). A low Q2 value of −0.314 and R2 = 0.907 for the 200 permutation tests indicated that the model was not overfitted (Figure 6B). A total of 326 differential metabolites were identified, compared to controls, of which 82 were down‐regulated and 244 were up‐regulated in the plasma of children with ASD (Figure 6C and Table S5). Cluster analysis allowed them to distinguish ASD from controls. However, children with ASD risk genes and children without ASD risk were overlapping (Figure 6D). The levels of differential metabolites between the ASD_M and ASD_nM groups were positively correlated (R = 0.65, *p* < 0.001), while the correlations between them and CTR were both negatively correlated (Figure 6E–G).

These results suggest that the distribution characteristics of metabolites were similar between the ASD_M and ASD_nM groups and could be effectively distinguished from the control group when they were combined into one group. This was further supported by correlation analysis of metabolite levels.

### 2.5 Bioinformatics analysis of the differential metabolites

**FIGURE 7:**
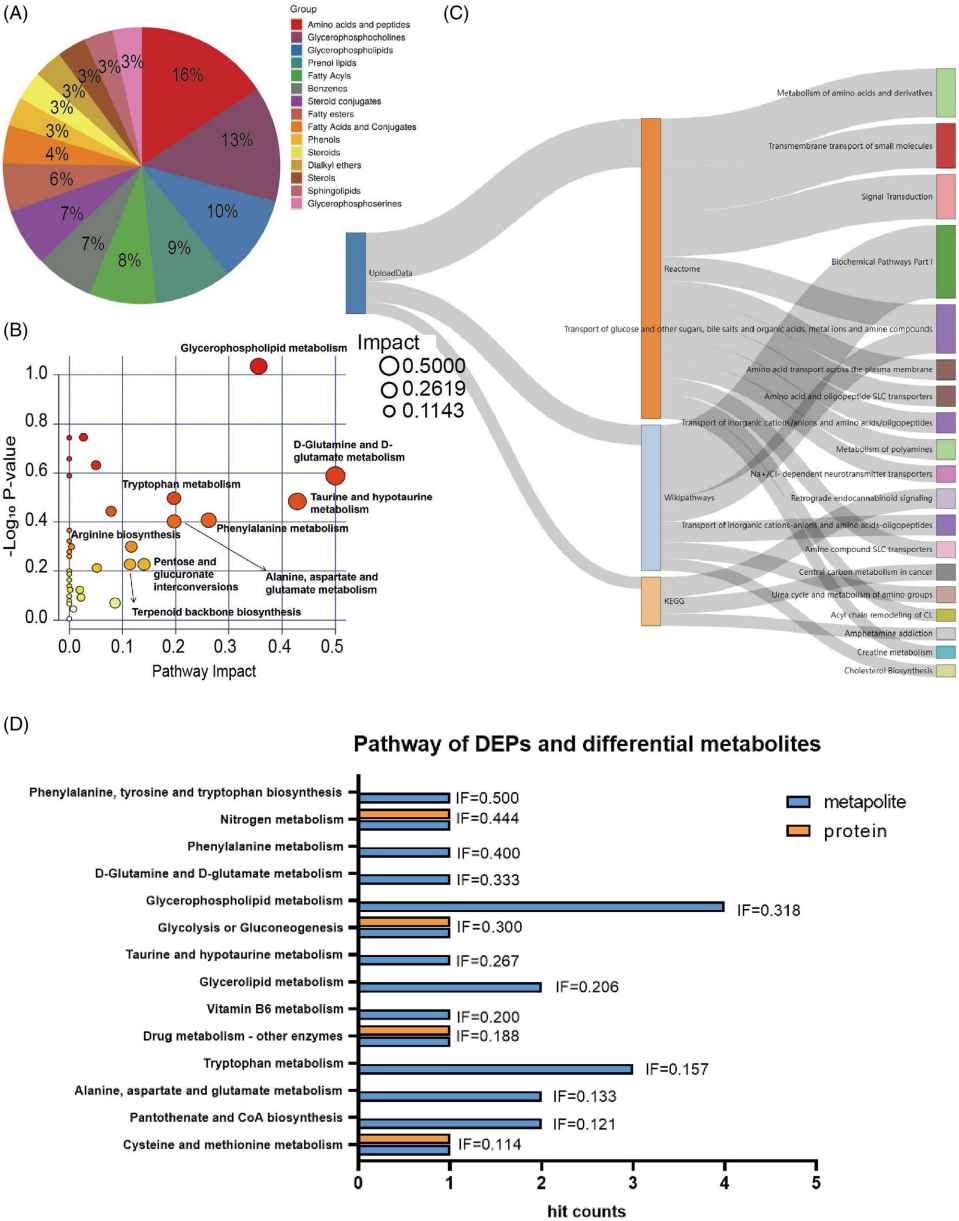
Bioinformatics analysis of differential metabolism between children with ASD and controls. (A) The differential metabolites were classified according to their chemical structure. (B) KEGG pathway analysis of differential metabolites. The size of the circle represents the size of the impact value. (C) Enrichment analysis of pathways associated with differential metabolites through REACTOME, Wiki, and KEGG pathway databases. (D) The integration analysis of DEPs and differential metabolites. Important KEGG analysis (*p* < 0.05 or pathway impact > 0.1). Impact factor (IF) indicates the impact value of the pathway. The higher the value of IF, the more important the pathway.

We then performed bioinformatic analysis of the differential metabolites. According to the chemical structure, the top three classes of differential metabolites were amino acids and peptides, glycerophosphocholines, and glycerophospholipids (Figure 7A). The KEGG pathways associated with them included glycerophospholipid metabolism, taurine and hypotaurine metabolism, D‐glutamine and D‐glutamate metabolism, phenylalanine metabolism, alanine, aspartate and glutamate metabolism, tryptophan metabolism, pentose and glucuronate interconversions, arginine biosynthesis, and terpenoid backbone biosynthesis were enriched (Figure 7B and Table 1; for these pathways, *p* < 0.05 and/or pathway impact > 0.1).

**TABLE 1:** The pathways associated with the differential metabolites.

Pathway name	Component ratio	*p*‐value	Impact	Hit compound
Glycerophospholipid metabolism	4/36	0.0025	0.3562	PE(O‐18:1(1Z)/20:4(5Z,8Z,11Z,14Z)), PC(18:3(6Z,9Z,12Z)/22:6(4Z,7Z,10Z,13Z,16Z,19Z)), lysoPC(22:0), lysoPA(0:0/18:2(9Z,12Z))
Glycerolipid metabolism	2/16	0.0281	0.0265	TG(12:0/12:0/12:0), lysoPA(0:0/18:2(9Z,12Z))
Tryptophan metabolism	3/41	0.0293	0.1969	L‐tryptophan, tryptamine, 5‐hydroxykynurenamine
Pantothenate and CoA biosynthesis	2/19	0.0388	0.0385	Pantetheine, pantothenic acid
Phenylalanine, tyrosine, and tryptophan biosynthesis	1/4	0.0655	0.0000	Phenylpyruvic acid
Alanine, aspartate, and glutamate metabolism	2/28	0.0783	0.1971	2‐Keto‐glutaramic acid, L‐glutamic acid
Linoleic acid metabolism	1/5	0.0812	0.0000	PC(14:0/20:2(11Z,14Z))
Porphyrin and chlorophyll metabolism	2/30	0.0883	0.0000	L‐glutamic acid, D‐urobilinogen
Nitrogen metabolism	1/6	0.0967	0.0000	L‐glutamic acid
D‐glutamine and D‐glutamate metabolism	1/6	0.0967	0.5000	L‐glutamic acid
Taurine and hypotaurine metabolism	1/8	0.1268	0.4286	Taurine
Vitamin B6 metabolism	1/9	0.1416	0.0784	Pyridoxamine
Steroid biosynthesis	2/42	0.1550	0.0516	24‐Hydroxycalcitriol, 7‐dehydrocholesterol
Phenylalanine metabolism	1/10	0.1561	0.2619	Phenylpyruvic acid
Aminoacyl‐tRNA biosynthesis	2/48	0.1912	0.0000	L‐tryptophan, L‐glutamic acid
Alpha‐linolenic acid metabolism	1/13	0.1981	0.0000	PC(18:3(6Z,9Z,12Z)/22:6(4Z,7Z,10Z,13Z,16Z,19Z))
Glycosylphosphatidylinositol‐anchor biosynthesis	1/14	0.2117	0.0040	PE(O‐16:1(1Z)/22:6(4Z,7Z,10Z,13Z,16Z,19Z))
Arginine biosynthesis	1/14	0.2117	0.1168	L‐glutamic acid
Butanoate metabolism	1/15	0.2250	0.0000	L‐glutamic acid
Histidine metabolism	1/16	0.2381	0.0000	L‐glutamic acid
Terpenoid backbone biosynthesis	1/18	0.2638	0.1143	Mevalonic acid
Pentose and glucuronate interconversions	1/18	0.2638	0.1406	Cholesterol glucuronide
Selenocompound metabolism	1/20	0.2885	0.0000	Selenocystathionine
Sphingolipid metabolism	1/21	0.3006	0.0000	SM(d18:0/16:1(9Z))
Glycolysis/gluconeogenesis	1/26	0.3582	0.0002	D‐glucose
Phosphatidylinositol signaling system	1/28	0.3799	0.0015	LysoPA(0:0/18:2(9Z,12Z))
Glutathione metabolism	1/28	0.3799	0.0197	L‐glutamic acid
Glyoxylate and dicarboxylate metabolism	1/32	0.4212	0.0000	L‐glutamic acid
Cysteine and methionine metabolism	1/33	0.4312	0.0218	3‐Mercaptopyruvic acid
Arachidonic acid metabolism	1/36	0.4599	0.0000	PC(14:0/20:2(11Z,14Z))
Arginine and proline metabolism	1/38	0.4783	0.0860	L‐glutamic acid
Fatty acid degradation	1/39	0.4873	0.0000	L‐palmitoylcarnitine
Primary bile acid biosynthesis	1/46	0.5461	0.0076	Taurine
Steroid hormone biosynthesis	1/85	0.7721	0.0000	Estriol‐16‐glucuronide

Through the analysis of multiple databases (KEGG, REACTOME, and Wiki pathways databases), the *p*‐value of 11 pathways was less than 0.05, and the hit components were greater than 5 (Figure 7C and Table S6). Of note, there were two SLC‐related transport pathways that were enriched. The correlation analysis of these differential metabolites is shown in Figure S2A, and the top 20 metabolites by metabolite–metabolite interaction are shown in Figure S2B and Table S4. Fifteen of them belonged to differential metabolites, which are shown in bold in Table S5. The first four compounds are L‐glutamic acid, L‐tryptophan, D‐glucose, and taurine.

### 2.6 Integrative analysis revealed key signaling pathways, DEPs, and differential metabolites

Integrative analysis of DEPs and differential metabolites can screen out key proteins, metabolites, and their associated mechanisms. Thus, we performed an integrative analysis of proteomics and metabolomics results. By integration analysis (Figure 7D), a total of 14 pathways were defined as critical pathways (*p* < 0.05 or pathway impact > 0.1). Among them, four pathways were simultaneously enriched DEPs and differential metabolites, including nitrogen metabolism, glycolysis or gluconeogenesis, drug metabolism‐other enzymes, and cysteine and methionine metabolism and their associated DEPs and differential metabolites, which were CA1 and L‐glutamic acid, BPGM and D ‐glucose, MDH1 and L‐glutamic acid, and MDH1 and 3‐mercaptopyruvic acid. Besides, MDH1 and L‐glutamic acid were repeatedly enriched several times. The results suggest that these pathways, DEPs, or differential metabolites may be closely related to ASD.

## 3 DISCUSSION

In this study, plasma proteomics and metabolomics analyses were performed on children with risk genes, children without risk genes, and healthy controls. As far as total plasma proteins and total plasma metabolites were concerned, there was little difference between the three groups. However, the protein or metabolism profile of the children with or with no risk genes was more clustered and overlapped, with a separation trend from the control. This trend is even more pronounced for the metabolic profile, implying that blood metabolites may favorably reflect the overall situation. This may be related to its smaller molecular weight and higher identified quantity. Of note, the similarity between the ASD_M group and the ASD_nM group suggests that the clinical diagnosis of ASD has a certain accuracy. However, for children who have not been detected for risk genes, the possibility that they are detected to carry the risk gene or have other genetic risks cannot be ruled out.

There were 41 DEPs identified in plasma proteomics analysis between the ASD and control groups. Previous studies have reported that many GO terms and pathways are associated with ASD, including the following categories: complement activation,^24^, ^25^ complement and coagulation cascades^23^, ^24^, ^25^; immune response^24^, ^25^; cell adhesion,^24^, ^26^ extracellular matrix organization,^27^ ECM‐receptor interaction,^27^ integrin cell surface interactions,^28^ and focal adhesion^24^, ^26^, ^29^; platelet degranulation and activation^30^; PI3K‐Akt signaling pathway.^31^

Among them, complement and immune‐related pathways have also been enriched in our previous plasma proteomic study of these five children with risk genes.^22^ The alteration in C2 was consistent with this study. In this study, most complement pathway proteins were upregulated (C2, CPB2, IGHV3‐74, and IGHV5‐51), supporting the notion that autistic patients may experience complement activation in their peripherals.^22^, ^25^, ^32^ In addition, research has emerged over the past 20 years that suggests that immune dysfunction is an important risk factor for ASD.^33^ Here, a total of 15 plasma DEPs were associated with the immune system. Interestingly, cell adhesion molecules, focal adhesion, and leukocyte transendothelial migration pathways have been associated with the Han Chinese cohort.^34^ Moreover,it has been shown that patients with ASD may experience gastrointestinal problems due to abnormal carbohydrate metabolism.^35^

The top 15 hub DEPs were involved in cell adhesion, cell motility, extracellular matrix organization, focal adhesion, PI3K‐Akt signaling pathway, and metabolism of carbohydrates, which further indicated that these several GO terms and pathways are important to ASD. Among them, in skeletal muscle, heart, brain, and creatine kinase isoenzyme (CKB) are essential in energy transduction in tissues with high and fluctuating energy demands. Similar to this study, it was observed to be increased in the prefrontal cortex in an autism‐like mouse model.^36^ Compared with CKB, three hub proteins (TNC, VTN, and TALDO1) were involved in more BPs and pathways. In the developing central nervous system, TNC regulates oligodendrocyte precursor cells and astrocyte proliferation.^37^ VTN is a cell adhesion and spreading factor present in serum and tissues and has a variety of functions.^24^, ^25^, ^38^ Contrary to this study, in the plasma of children with ASDs, it was found to be up‐regulated.^24^ Another protein, TALDO1, was up‐regulated in the plasma of children with ASD. The protein is highly expressed in the oligodendrocytes of the brain and has been linked to ASDs.^39^ Several other hub proteins are also of interest, including EGFR, CBP2 and MDH1. EGFR is essential to neural cell development and repair.^40^ CBP2 is one of the important proteins in the immune system and is involved in blood clotting. Similarly to our finding, EGFR^41^ and the peptide level of CBP2^42^ have been observed to be increased in the blood of children with ASD. MDH1 is important to the malate–aspartate shuttle and tricarboxylic acid cycle as well as in the oxidative phosphorylation of mitochondrial NADH supply. Here, the reduced level of MDH1 in ASD plasma implies mitochondrial dysfunction in children with ASD.^43^

According to metabolomic analysis, differential metabolites were mostly involved in amino acid and glycerophospholipid metabolism. These differential metabolites and their associated pathways have previously been reported to be associated with ASD.^8^, ^22^, ^44^ There are three amino acids that are essential to the development of ASD,that is, L‐glutamic acid, L‐tryptophan, and taurine. Previous studies have shown the altered levels of glutamate and glutamine in the blood of autistic patients.^8^, ^45^, ^46^ There is a significant reduction in glutamatergic signals in the anterior cingulate cortex and cerebellum of patients with ASD.^47^, ^48^ The results support the theory that ASD is caused by excitatory/inhibitory imbalances, particularly in glutamate neurotransmitter.^49^

Recently, ASD children have exhibited significant reductions in gut metabolites associated with glutamate and tryptophan metabolism,^50^ which is linked to variations in gut bacteria involved in the metabolism of D‐glutamine and D‐glutamate, indicating that an abnormal glutamate metabolism may be caused by the gut microbiota in children with autism.^51^ Taurine^8^, ^52^ and tryptophan^8^ levels have been observed to change in the blood and urine of children with ASD. As far as we know, this is the first time to find that the plasma D‐glucose increases in the plasma of children with ASD. These preliminary findings suggest that children with ASD have a number of common metabolic disorders and highlight the importance of examining metabolites in various biological samples in more detail. The analysis of gut microbes and the detection of microbial‐derived metabolites in feces, as well as metabolites originating from gut microbes in blood and urine, are topics of interest for future research.^53^

The brain is particularly rich in lipids and has a different lipid composition.^54^ Disturbance of glycerophospholipid metabolism has been observed in autistic mice^55^ and the plasma of children with ASD.^22^, ^56^ Our results support that the metabolism of glycerophospholipids in plasma was dysregulated in children with ASD. We also noted that SLC‐related pathways were enriched in different databases. There are many different types of transmembrane transporters, but solute carriers (SLCs) are the most common ones.^57^ Transporters are involved in many physiological processes in the brain, such as energy metabolism, glutamate/γ aminobutyric acid‐glutamine circulation, and maintenance of blood–brain barrier function.^57^ Mutations in some transporters have been shown to be associated with ASD, such as SLC7A5, SLC19A1, and SLC25A12 genes.^57^, ^58^ Results indicate that this mechanism may contribute to ASD pathogenesis.

Finally, integrating omics data, the DEPs and differential metabolites were enriched to nitrogen metabolism, glyoxylate and dicarboxylate metabolism, glycolysis or gluconeogenesis, and cysteine and methionine metabolism. The differential metabolites involved were L‐glutamic acid, D‐glucose, and 3‐mercaptopyruvic acid, while the proteins were CA1, MDH1, and BPGM. L‐glutamic acid and MDH1 were repeatedly enriched, which may be important protein or metabolite in the pathogenesis of ASD.

**FIGURE 8:**
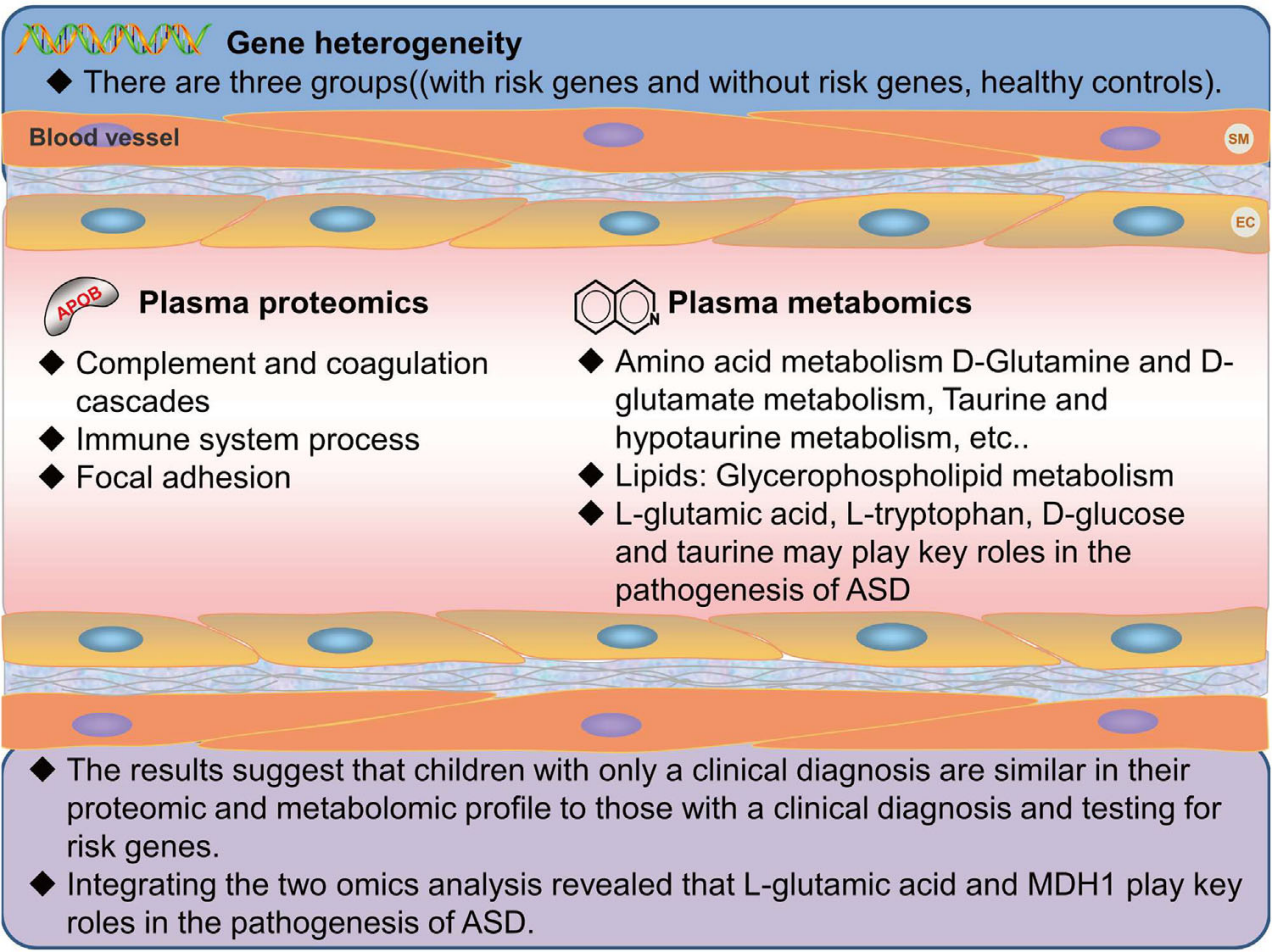
Common mechanisms and potential biomarker obtained from the children with or without ASD risk gene and healthy controls in this study. Bold letters indicate that these pathways are associated with the DEPs and differential metabolites in this study. Abbreviations: MDH1, malate dehydrogenase.

In conclusion, the results of this study suggest that despite genetic heterogeneity among children with ASD, it is still possible to find differential proteins or differential metabolites between ASD and controls that can distinguish the two groups. The mechanisms associated with these differential proteins or metabolites are focused on several previously reported mechanisms or signaling pathways (Figure 8), suggesting that they may be potential diagnostic markers. In line with our previous study,^22^ these results support the possibility of finding different “commonalities” in blood proteins and metabolites between children with ASD and controls as diagnostic markers. It is therefore important to further elucidate the mechanisms and pathways that focus on blood DEPs and differential metabolites between children with ASD and controls, as well as which proteins or metabolites are more frequently reported and have consistent changes, and to investigate them. Indeed, studies of ASD metabolites and proteins as diagnostic markers have recently been reviewed.^8^, ^9^, ^10^, ^59^, ^60^, ^61^ Our results also suggest that children with only a clinical diagnosis are similar in their proteomic and metabolomic profile to those with a clinical diagnosis and testing for risk genes. Although further confirmation is needed, the likelihood of finding diagnostic markers has increased. It is important to note that this is only a preliminary study, and there is still a need to expand the sample size and increase the number of children with ASD carrying different types of risk genes to be studied. Besides, the differential expression of some important DEPs or differential metabolites among different groups deserves further validation.

## 4 MATERIALS AND METHODS

### 4.1 Plasma samples

In Shenzhen, China, 122 children with ASD were recruited, who were 2−6 years old from Baoan Maternal and Child Health Hospital. In the previous section, we described the diagnostic and exclusion criteria for ASD.^22^, ^23^ Briefly, diagnosis was made according to DSM‐V criteria, and ABC and CARS scale assessments were also performed. After genetic testing, five children carrying the risk gene were set as the ASD_M group; five children without the risk gene were randomly selected as the ASD_nM group for proteomic and metabolomic analysis; and 10 age‐ and sex‐matched healthy controls were selected as the CTR group for proteomic and metabolomic analysis, respectively. Plasma samples were collected as previously described.^22^ When selecting samples, we are all randomly matched and selected.

### 4.2 Proteomics analysis of plasma

#### 4.2.1 Protein sample preparation

The top 14 most abundant proteins of plasma were depleted by a Multi Affinity Removal Columns, Human‐14 (Agilent Technologies) from plasma. A filter‐aided sample preparation method was employed in‐solution protein digestion.^62^ Approximately 100 μg of proteins from each sample were reduced, alkylated, and digested with trypsin (Promega) as described previously.^63^, ^64^ Centrifugation was used to separate the peptides for 15 min at 12,000 g, 4°C. Following reverse‐phase chromatography (Durashell, C18, 250 × 4.6 mm, 5 μm; Bonna‐Agela Technologies Inc.) at high pH, the peptides (400 μg) were subjected to salt removal using an Agilent HPLC system (Agilent Technologies). The peptides were then lyophilized and kept at 80°C after being eluted and mixed into 30 batches.^65^

#### 4.2.2 Plasma proteomics analysis based on SWATH‐MS technology

SWATH‐MS analysis was performed as described in previous studies.^63^, ^64^ In information‐dependent acquisition (IDA) analysis, 3 μg peptide of each batch was injected for eluting that used a gradient elution with solvents A and B at 5 μL/min for 60 min. Based on IDA raw data, 75 variable Q1 isolation window scheme was calculated by SWATH Variable Window Assay Calculator V1.1. Data‐independent acquisition was performed using SWATH method, such as analyzing 1 μg peptide of each sample under the same chromatographic conditions as the IDA library sample. After the acquisition, the IDA data files were analyzed using ProteinPilot (version 5.0, ) in thorough mode using the Paragon algorithm.

#### 4.2.3 SWATH‐MS data analysis and bioinformatics analysis

Further statistical analysis of the quality control normalized peak intensities was performed using the StatTarget R package.^66^ SIMCA‐P (V14.1, Sartorius Stedim Data Analytics AB) was used to perform PCA, PLS‐DA, and OPLS‐DA. VIP (variable importance in projection) score was obtained from OPLS‐DA. The model was validated with a seven‐fold cross‐validation method and 200 random permutations. Quantitative data were loaded into the OMICSBEAN (http://www.omicsbean.cn) database for analysis. Compared to control, proteins with fold change (FC) ≥ 1.2 or FC ≤ 0.83, VIP scores ≥ 1, and *p*‐values < 0.05 were identified as DEPs.^67^ BP, CC, KEGG, REACTOME pathway, and Wiki pathway analyses of DEPs were performed using the String database (version 9.1, http://string‐db.org/). Cytoscape was used to analyze protein–protein interaction networks.

### 4.3 Plasma metabolomics analysis

#### 4.3.1 Metabolomics MS analysis

Plasma metabolomics studies were performed as previously described.^22^ For each sample, metabolites were extracted from 100 μL of plasma.^22^ Add 0.3 mg of internal standard (L‐2‐chlorophenylalanine (HEPENG BIOTECHNOLOGY, 99%) per 1 mL of sample. A metabolite extract was prepared using methanol (MACKLIN, 99.5%) and acetonitrile (ACMEC, 99.9%) (2:1, v/v), which was pre‐cooled to −20°C.^68^

A Waters Acquity UPLC system coupled with Waters xevo g2 xs (Waters) was used for metabolomics analysis. This gradient elution is illustrated in Table S7. The injection volume was 2 μL. The parameters of MS analysis are shown in Table S8. Raw spectra were processed using Progenesis QI 2.0 software (Nonlinear Dynamics) for peak selection, comparison, normalization, and identification. The public spectrum libraries MassBank, HMDB, LipidBlast, and METLIN were used for secondary identification.

#### 4.3.2 Metabolomics data analysis

The normalized peak intensity was further analyzed with StatTarget R package.^66^ The results of anion and cation modes were combined, and multivariate statistical analysis was performed.^69^ PCA, PLS‐DA, and OPLS‐DA were performed as described above. The metabolites with FC ≥ 1.5 or ≤ 0.67 (ASD vs. control group), VIP scores ≥ 1, and *p* < 0.05 were identified as differential metabolites. Pathway analysis was performed by using Omicsolution (https://www.omicsolution.org/) and MetaboAnalyst 5.0 (https://www.metaboanalyst.ca).^70^

### 4.4 Integrated analysis of DEPs and differential metabolites identified in this study

For integrated pathway analysis, DEPs from proteome analysis of the patients and controls and differential metabolites from plasma metabolomics of the two groups were put into MetaboAnalyst 5.0 (https://www.metaboanalyst.ca).^70^

### 4.5 Statistical analysis

SPSS software (version 24.0, International Business Machines Corporation) was used for statistical analysis of the data. The statistical analysis is first based on the Kolmogorov–Smirnov test for the distribution of values. These data were normally distributed, and the two‐tailed independent samples *t*‐test was chosen for statistical assessment, which was performed. Pearson correlation analysis was performed using ggpubr R package to analyze the correlation of total or differential protein expression levels and total or differential metabolite levels between the two groups of the three groups (ASD_M, ASD_nM, and CTR). *p* < 0.05 was considered significant for the correlation between the groups.

## AUTHOR CONTRIBUTIONS

L.M.S. conceived and designed the experiments; X.X.T., Y.X.Z., H.J.Z., X.S.C., J.L., H.B.Z., Y.Y.F., and H.H.W. performed the experiments and analyzed the data; L.M.S. and X.X.T. wrote, reviewed, and edited the manuscript. Y.G., Q.H., and C.Y.F. carried out the clinical studies and collected the blood samples. All authors have read and approved the final manuscript.

## CONFLICT OF INTEREST STATEMENT

The authors declare no conflicts of interest.

## ETHICS STATEMENT

The research was approved by Human Research Ethics Committees of Shenzhen University (No. M20220203) and Maternal and Child Health Hospital of Baoan (No. 20170801) and complies with the guidelines of the Helsinki Declaration. Written informed consent for study participation was obtained from the children's guardian.

## Supporting information

Supporting Information

## Data Availability

The data that support the findings of this study are available from the corresponding author upon reasonable request.
