# Supplementary material for: A study of genetic heterogeneity in autism spectrum disorders based on plasma proteomic and metabolomic analysis: multiomics study of autism heterogeneity
Source: MedComm (2020). 2023 Sep 24;4(5):e380. doi: 10.1002/mco2.380 (PMC10518435; doi:10.1002/mco2.380)
Supplement: Supplementary file 1 — Supporting Information [file MCO2-4-e380-s001.docx]

**A study of genetic heterogeneity in autism spectrum disorders based on plasma proteomic and metabolomic analysis**

**Multiomics study of autism heterogeneity**

Xiaoxiao Tang^1^, Chengyun Feng^2^，Yuxi Zhao^1^，Huajie Zhang^1^, Yan Gao^2^，Xueshan Cao^1^, Qi Hong^2^, Jing Lin^1^, Hongbin Zhuang^1^, Yuying Feng^1^, Hanghang Wang^1^, Liming Shen^1, 3, 4^*

1 College of Life Science and Oceanography, Shenzhen University, Shenzhen 518071

P. R. China

2 Maternal and Child Health Hospital of Baoan, Shenzhen 518100, PR China

3 Shenzhen-Hong Kong Institute of Brain Science-Shenzhen Fundamental Research

Institutions, Shenzhen 518055, P. R. China

4 Shenzhen Key Laboratory of Marine Biotechnology and Ecology, Shenzhen 518071, P. R. China

**Corresponding Author:**

Liming Shen

College of Life Sciences and Oceanography, Shenzhen University, Shenzhen 518060,

P. R. China

E-mail: slm@szu.edu.cn

# Supplement tables

**Table S1. The statistic analysis of children with ASD and their corresponding healthy control information in this study.**

|  |  | ASD_M | ASD_nM | *P* value | total ASD | CTR | *P* value |
| --- | --- | --- | --- | --- | --- | --- | --- |
| Proteomics samples | Samples | 5 | 5 | - | 10(8 males and 2 females) | 10(8 males and 2 females) | - |
|  | BMI | 14.3±1.01 | 15.2±0.68 | 0.2081 | 14.8±0.97 | 15.3±1.19 | 0.4146 |
|  | Age in months | 49.0±21.32 | 46.8±21.74 | 0.7001 | 47.9±21.56 | 38.8±11.29 | 0.2766 |
|  | ABC | 81.0±11.50 | 73.0±25.66 | 0.6729 | 77.0±20.30 | - | - |
|  | CARS | 37.0±1.02 | 38.0±3.66 | 0.5826 | 37.2±2.75 | - | - |
| Metabolomics samples | Samples | 5 | 5 | - | 10(8 males and 2 females) | 10(8 males and 2 females) | - |
|  | BMI | 14.3±1.01 | 15.3±1.20 | 0.3831 | 14.8±1.20 | 15.4±0.60 | 0.1412 |
|  | Age in months | 49.0±21.32 | 49.0±21.14 | 1.0000 | 49.0±21.23 | 49.8±19.98 | 0.9353 |
|  | ABC | 81.0±11.50 | 62.0±23.86 | 0.3338 | 72.0±20.96 | - | - |
|  | CARS | 37.0±1.02 | 37.0±2.99 | 0.9002 | 36.7±2.24 | - | - |

The CARS scores for the single items are added together into a total score, which classifies the child as not autistic (below 30), mild or moderately autistic (30-36.5), or severely autistic (above 36.5). A CARS score of 30 or higher was used as threshold value for diagnosis of autism. A ABC score of 53 was used as threshold value for diagnosis of autism. (Meng et al., Neurosci Bull 2018, 34: 789-800)

**Table S2. The children with ASD and their corresponding healthy control information in this study.**

| Sample | Age (month) | Gender | ABC | CARS | Ref. | Alt. | AAChange. refgene | ASD risk gene testing results |
| --- | --- | --- | --- | --- | --- | --- | --- | --- |
| 23A-ASH1L | 23 | male | 79 | 38 | T | G | ASH1L:NM_018489:exon16:c.A7058C:p.N2353T | de novo mutation in ASH1L gene |
| 28A-SCN2A | 28 | male | 67 | 36 | G | A | SCN2A:NM_001040143:exon20:c.G3956A:p.R1319Q; | de novo mutation in SCN2A gene |
| 48A-GIGYF2 | 48 | male | 73 | 37 | GATA | - | GIGYF2:NM_001103148:exon10:c.1060_1063del:p.D354fs; | de novo mutation in GIGYF2 gene |
| 72A-DDX3X | 72 | female | 100 | 35 | C | T | DDX3X: NM_001356: exon 6: c.452 dup T: p. F151fs | de novo mutation in DDX3X gene |
| 74A-NAA15 | 74 | male | 87 | 37 | G | - | NAA15:NM_057175:exon17:c.2061delG:p.K687fs | de novo mutation in NAA15 gene |

**Table S3. Differentially expressed protein between total ASD and the CTR group by SWATH Analysis.**

| Accession no. | Protein name | Uniprot ID | Gene name | Fold change ^†^ | *P* value | VIP |  |
| --- | --- | --- | --- | --- | --- | --- | --- |
| 1 | Immunoglobulin heavy variable 3-74 | A0A0B4J1X5 | IGHV3-74 | 1.37 | 0.0093 | 1.75 |  |
| 2 | Immunoglobulin heavy variable 5-51 | A0A0C4DH38 | IGHV5-51 | 1.32 | 0.0326 | 1.62 |  |
| 3 | FYN-binding protein 1 | O15117 | FYB1 | 0.76 | 0.0277 | 1.37 |  |
| 4 | Actin-related protein 2/3 complex subunit 3 | O15145 | ARPC3 | 0.74 | 0.0219 | 1.72 |  |
| 5 | Sushi domain-containing protein 5 | O60279 | SUSD5 | 1.28 | 0.0146 | 1.91 |  |
| 6 | WD repeat-containing protein 1 | O75083 | WDR1 | 0.70 | 0.0485 | 1.56 |  |
| 7 | Ficolin-3 | O75636 | FCN3 | 0.71 | 0.0233 | 1.50 |  |
| 8 | Reversion-inducing cysteine-rich protein with Kazal motifs | O95980 | RECK | 1.34 | 0.0168 | 1.96 |  |
| 9 | Hypoxanthine-guanine phosphoribosyltransferase | P00492 | HPRT1 | 1.29 | 0.0156 | 1.81 |  |
| 10 | Epidermal growth factor receptor | P00533 | EGFR | 8.74 | 0.0010 | 1.69 |  |
| 11 | Carbonic anhydrase 1 | P00915 | CA1 | 0.76 | 0.0270 | 1.60 |  |
| 12 | Alpha-1-antichymotrypsin | P01011 | SERPINA3 | 2.46 | 0.0494 | 1.11 |  |
| 13 | Immunoglobulin kappa variable 1-5 | P01602 | IGKV1-5 | 0.73 | 0.0263 | 1.66 |  |
| 14 | Immunoglobulin lambda variable 1-40 | P01703 | IGLV1-40 | 0.57 | 0.0005 | 2.18 |  |
| 15 | Hemoglobin subunit delta | P02042 | HBD | 2.00 | 0.0178 | 1.60 |  |
| 16 | Beta-2-glycoprotein 1 | P02749 | APOH | 1.84 | 0.0027 | 2.12 |  |
| 17 | Platelet basic protein | P02775 | PPBP | 0.73 | 0.0121 | 1.63 |  |
| 18 | Vitronectin | P04004 | VTN | 0.44 | 0.0000 | 2.34 |  |
| 19 | Alpha-1B-glycoprotein | P04217 | A1BG | 0.76 | 0.0211 | 1.69 |  |
| 20 | Sex hormone-binding globulin | P04278 | SHBG | 0.80 | 0.0422 | 1.52 |  |
| 21 | Pancreatic alpha-amylase | P04746 | AMY2A | 0.75 | 0.0288 | 1.90 |  |
| 22 | Cholinesterase | P06276 | BCHE | 1.75 | 0.0469 | 1.25 |  |
| 23 | Complement C2 | P06681 | C2 | 1.33 | 0.0096 | 2.09 |  |
| 24 | Asialoglycoprotein receptor 2 | P07307 | ASGR2 | 1.84 | 0.0304 | 1.50 |  |
| 25 | Calpain-1 catalytic subunit | P07384 | CAPN1 | 0.59 | 0.0120 | 1.69 |  |
| 26 | Beta-hexosaminidase subunit beta | P07686 | HEXB | 0.82 | 0.0136 | 1.68 |  |
| 27 | Bisphosphoglycerate mutase | P07738 | BPGM | 1.63 | 0.0490 | 1.19 |  |
| 28 | Integrin alpha-5 | P08648 | ITGA5 | 0.60 | 0.0357 | 1.43 |  |
| 29 | Creatine kinase B-type | P12277 | CKB | 2.32 | 0.0000 | 2.73 |  |
| 30 | Versican core protein | P13611 | VCAN | 2.28 | 0.0496 | 1.61 |  |
| 31 | Liver carboxylesterase 1 | P23141 | CES1 | 1.47 | 0.0234 | 1.85 |  |
| 32 | Tenascin | P24821 | TNC | 0.78 | 0.0445 | 1.27 |  |
| 33 | Intercellular adhesion molecule 3 | P32942 | ICAM3 | 0.60 | 0.0149 | 1.73 |  |
| 34 | Transaldolase | P37837 | TALDO1 | 2.74 | 0.0009 | 1.99 |  |
| 35 | Malate dehydrogenase, cytoplasmic | P40925 | MDH1 | 0.36 | 0.0198 | 1.63 |  |
| 36 | IgG receptor FcRn large subunit p51 | P55899 | FCGRT | 0.75 | 0.0210 | 1.64 |  |
| 37 | Carboxypeptidase B2 | Q96IY4 | CPB2 | 1.30 | 0.0315 | 1.57 |  |
| 38 | NIF3-like protein 1 | Q9GZT8 | NIF3L1 | 1.49 | 0.0252 | 1.74 |  |
| 39 | Aminopeptidase B | Q9H4A4 | RNPEP | 2.34 | 0.0143 | 1.92 |  |
| 40 | Thrombospondin-3 | F5H4Z8 | THBS3 | 0.13 | 0.0167 | 1.66 |  |
| 41 | CD44 antigen | H0YD13 | CD44 | 2.22 | 0.0415 | 1.76 |  |

†: *P* < 0.05 vs. the control

**Table S4. Enrichment analysis by different databases.**

| Database | Term ID | Term description | Observed gene count | Background gene count | Strength | False discovery rate | Matching proteins in network (labels) |
| --- | --- | --- | --- | --- | --- | --- | --- |
| BP | GO:0002376 | Immune system process | 17 | 2481 | 0.56 | 0.003 | ICAM3, FCGRT, VTN, ARPC3, HEXB, A1BG, FCN3, ITGA5, PPBP, HPRT1, C2, GIG25, BPGM, CD44, FYB, WDR1, CAPN1 |
| BP | GO:0016192 | Vesicle-mediated transport | 15 | 1805 | 0.64 | 0.003 | ICAM3, APOH, FCGRT, VTN, ARPC3, HEXB, A1BG, FCN3, PPBP, ASGR2, CES1, GIG25, CD44, WDR1, CAPN1 |
| BP | GO:0002252 | Immune effector process | 11 | 969 | 0.78 | 0.003 | ARPC3, HEXB, A1BG, FCN3, PPBP, HPRT1, C2, GIG25, CD44, WDR1, CAPN1 |
| BP | GO:0002443 | Leukocyte mediated immunity | 9 | 641 | 0.87 | 0.005 | HEXB, A1BG, PPBP, HPRT1, C2, GIG25, CD44, WDR1, CAPN1 |
| BP | GO:0030198 | Extracellular matrix organization | 8 | 338 | 1.10 | 0.003 | ICAM3, VTN, VCAN, TNC, ITGA5, RECK, CD44, CAPN1 |
| BP | GO:0042060 | Wound healing | 7 | 439 | 0.93 | 0.028 | CPB2, APOH, TNC, EGFR, ITGA5, HBD, CD44 |
| BP | GO:0002576 | Platelet degranulation | 5 | 129 | 1.31 | 0.009 | APOH, A1BG, PPBP, GIG25, WDR1 |
| CC | GO:0005615 | Extracellular space | 30 | 3195 | 0.70 | 0.000 | ICAM3, CPB2, APOH, FCGRT, VTN, ARPC3, HEXB, A1BG, BCHE, VCAN, TNC, FCN3, EGFR, RNPEP, PPBP, HPRT1, CKB, C2, TALDO1, CES1, HBD, SHBG, GIG25, BPGM, CD44, WDR1, CA1, CAPN1, MDH1, AMY2A |
| CC | GO:0005576 | Extracellular region | 32 | 4166 | 0.61 | 0.000 | ICAM3, CPB2, APOH, FCGRT, VTN, ARPC3, HEXB, A1BG, BCHE, VCAN, TNC, FCN3, EGFR, RNPEP, PPBP, HPRT1, CKB, C2, TALDO1, CES1, THBS3, RECK, HBD, SHBG, GIG25, BPGM, CD44, WDR1, CA1, CAPN1, MDH1, AMY2A |
| CC | GO:0070062 | Extracellular exosome | 21 | 2099 | 0.72 | 0.000 | ICAM3, CPB2, APOH, VTN, ARPC3, HEXB, A1BG, RNPEP, HPRT1, CKB, C2, TALDO1, SHBG, GIG25, BPGM, CD44, WDR1, CA1, CAPN1, MDH1, AMY2A |
| CC | GO:0031982 | Vesicle | 25 | 3879 | 0.53 | 0.000 | ICAM3, CPB2, APOH, FCGRT, VTN, ARPC3, HEXB, A1BG, EGFR, ITGA5, RNPEP, PPBP, HPRT1, CKB, C2, TALDO1, SHBG, GIG25, BPGM, CD44, WDR1, CA1, CAPN1, MDH1, AMY2A |
| CC | GO:0072562 | Blood microparticle | 6 | 115 | 1.44 | 0.000 | VTN, A1BG, BCHE, FCN3, HBD, GIG25 |
| CC | GO:0062023 | Collagen-containing extracellular matrix | 8 | 396 | 1.03 | 0.000 | APOH, VTN, A1BG, VCAN, TNC, FCN3, THBS3, GIG25 |
| CC | GO:0031983 | Vesicle lumen | 6 | 330 | 0.98 | 0.006 | APOH, HEXB, A1BG, EGFR, PPBP, GIG25 |
| CC | GO:0071062 | alphav-beta3 integrin-vitronectin complex | 2 | 3 | 2.55 | 0.006 | VTN, ITGA5 |
| CC | GO:0005925 | Focal adhesion | 6 | 405 | 0.89 | 0.016 | ARPC3, TNC, EGFR, ITGA5, CD44, CAPN1 |
| CC | GO:0030141 | Secretory granule | 8 | 845 | 0.70 | 0.019 | APOH, HEXB, A1BG, RNPEP, PPBP, GIG25, CD44, CAPN1 |
| KEGG | hsa04512 | ECM-receptor interaction | 5 | 88 | 1.48 | 0.000 | VTN, TNC, ITGA5, THBS3, CD44 |
| KEGG | hsa05206 | MicroRNAs in cancer | 5 | 160 | 1.22 | 0.002 | TNC, EGFR, ITGA5, RECK, CD44 |
| KEGG | hsa04510 | Focal adhesion | 5 | 198 | 1.13 | 0.004 | VTN, TNC, EGFR, ITGA5, THBS3 |
| KEGG | hsa05131 | Shigellosis | 5 | 218 | 1.08 | 0.005 | ARPC3, EGFR, ITGA5, CD44, CAPN1 |
| KEGG | hsa05165 | Human papillomavirus infection | 5 | 325 | 0.91 | 0.025 | VTN, TNC, EGFR, ITGA5, THBS3 |
| KEGG | hsa04151 | PI3K-Akt signaling pathway | 5 | 350 | 0.88 | 0.029 | VTN, TNC, EGFR, ITGA5, THBS3 |
| KEGG | hsa05205 | Proteoglycans in cancer | 4 | 196 | 1.03 | 0.029 | VTN, EGFR, ITGA5, CD44 |
| KEGG | hsa04610 | Complement and coagulation cascades | 3 | 82 | 1.29 | 0.029 | CPB2, VTN, C2 |
| Reactome | HSA-168256 | Immune System | 15 | 1956 | 0.61 | 0.001 | ICAM3, CPB2, VTN, ARPC3, HEXB, A1BG, FCN3, PPBP, C2, TALDO1, GIG25, CD44, FYB, CA1, CAPN1 |
| Reactome | HSA-168249 | Innate Immune System | 12 | 1025 | 0.79 | 0.001 | ICAM3, CPB2, VTN, ARPC3, HEXB, A1BG, FCN3, PPBP, C2, GIG25, CD44, CAPN1 |
| Reactome | HSA-109582 | Hemostasis | 8 | 605 | 0.84 | 0.004 | APOH, A1BG, ITGA5, PPBP, HBD, GIG25, CD44, WDR1 |
| Reactome | HSA-1474244 | Extracellular matrix organization | 7 | 301 | 1.09 | 0.001 | ICAM3, VTN, VCAN, TNC, ITGA5, CD44, CAPN1 |
| Reactome | HSA-71387 | Metabolism of carbohydrates | 6 | 292 | 1.04 | 0.004 | HEXB, VCAN, TALDO1, BPGM, CD44, MDH1 |
| Reactome | HSA-6798695 | Neutrophil degranulation | 6 | 473 | 0.83 | 0.046 | HEXB, A1BG, PPBP, GIG25, CD44, CAPN1 |
| Reactome | HSA-216083 | Integrin cell surface interactions | 5 | 85 | 1.49 | 0.001 | ICAM3, VTN, TNC, ITGA5, CD44 |
| Reactome | HSA-114608 | Platelet degranulation | 5 | 127 | 1.32 | 0.002 | APOH, A1BG, PPBP, GIG25, WDR1 |
| Reactome | HSA-166658 | Complement cascade | 4 | 57 | 1.57 | 0.002 | CPB2, VTN, FCN3, C2 |
| Reactome | HSA-977606 | Regulation of Complement cascade | 3 | 47 | 1.53 | 0.025 | CPB2, VTN, C2 |
| Wikipathways | WP2645 | Heroin metabolism | 2 | 3 | 2.55 | 0.023 | BCHE, CES1 |
| Wikipathways | WP2826 | Cocaine metabolism | 2 | 4 | 2.42 | 0.023 | BCHE, CES1 |
| Wikipathways | WP306 | Focal adhesion | 5 | 196 | 1.13 | 0.023 | VTN, TNC, EGFR, ITGA5, THBS3 |
| Wikipathways | WP3932 | Focal adhesion: PI3K-Akt-mTOR-signaling pathway | 5 | 302 | 0.94 | 0.045 | VTN, TNC, EGFR, ITGA5, THBS3 |
| Wikipathways | WP229 | Irinotecan pathway | 2 | 13 | 1.91 | 0.049 | BCHE,CES1 |
| Wikipathways | WP4172 | PI3K-Akt signaling pathway | 5 | 336 | 0.90 | 0.049 | VTN,TNC,EGFR, ITGA5, THBS3 |

**Table S5. Differential metabolites between total ASD and the CTR group.**

| Accession no. | HMDB ID | Compound Name | Fold change † | *P* value | VIP | Retention time (min) |
| --- | --- | --- | --- | --- | --- | --- |
| 1 | HMDB0034767 | (1R*,3R*,3'S*)-1,2,3,4-Tetrahydro-1-(2-thio-3-pyrrolidinyl)-beta-carboline-3-carboxylic acid | 0.66 | 0.0266 | 1.10 | 0.6120 |
| 2 | HMDB0126978 | (2-hydroxy-5-{5-hydroxy-4-oxo-6,8-bis[3,4,5-trihydroxy-6-(hydroxymethyl)oxan-2-yl]-7-[(3,4,5-trihydroxy-6-methyloxan-2-yl)oxy]-4H-chromen-2-yl}phenyl)oxidanesulfonic acid | 1.86 | 0.0003 | 1.74 | 0.6512 |
| 3 | HMDB0002430 | (3a,5b,7a)-23-Carboxy-7-hydroxy-24-norcholan-3-yl-b-D-Glucopyranosiduronic acid | 2.23 | 0.0071 | 1.49 | 4.8522 |
| 4 | HMDB0006537 | (a-D-mannosyl)2-b-D-mannosyl-N-acetylglucosamine | 1.64 | 0.0342 | 1.33 | 5.8649 |
| 5 | HMDB0036013 | (R)-3,4-Dihydro-2-(4,8,12-trimethyl-3,7,11-tridecatrienyl)-2H-1-benzopyran-6-ol | 1.53 | 0.0040 | 1.72 | 8.3879 |
| 6 | HMDB0039930 | (S)-Multifidol 2-[apiosyl-(1->6)-glucoside] | 1.55 | 0.0067 | 1.58 | 3.5288 |
| 7 | HMDB0128516 | {4-[(E)-2-(2,3,5-trihydroxyphenyl)ethenyl]phenyl}oxidanesulfonic acid | 2.27 | 0.0015 | 1.84 | 3.0892 |
| 8 | HMDB0002144 | 1,3-Dimethyluracil | 0.49 | 0.0271 | 1.35 | 0.6120 |
| 9 | HMDB0031923 | 10,20-Dihydroxyeicosanoic acid | 0.38 | 0.0058 | 1.43 | 8.4872 |
| 10 | 88174 | 12-Oxo-2,3-dinor-10,15-phytodienoic acid | 2.12 | 0.0263 | 1.31 | 5.6108 |
| 11 | HMDB0012553 | 12-Oxo-20-trihydroxy-leukotriene B4 | 0.36 | 0.0002 | 1.78 | 3.3166 |
| 12 | HMDB0012560 | 13'-Hydroxy-alpha-tocotrienol | 2.1 | 0.0363 | 1.34 | 6.3997 |
| 13 | HMDB0012561 | 13'-Hydroxy-gamma-tocopherol | 1.86 | 0.0002 | 1.94 | 9.7633 |
| 14 | 35229 | 17-Octadecynoic Acid | 0.4 | 0.0012 | 1.72 | 5.7730 |
| 15 | HMDB0012496 | 1-Lyso-2-arachidonoyl-phosphatidate | 0.43 | 0.0149 | 1.36 | 9.0211 |
| 16 | HMDB0034857 | 2-(2-Thienylmethylene)-1,6-dioxaspiro[4.4]non-3-ene | 0.63 | 0.0097 | 1.36 | 0.7872 |
| 17 | HMDB0006228 | 24-Hydroxycalcitriol | 1.52 | 0.0203 | 1.44 | 5.6031 |
| 18 | HMDB0002157 | 27-Norcholestanehexol | 16.26 | 0.0027 | 1.65 | 5.9894 |
| 19 | HMDB0001552 | 2-Keto-glutaramic acid | 0.54 | 0.0096 | 1.44 | 11.8077 |
| 20 | HMDB0000339 | 2-Methylbutyrylglycine | 1.53 | 0.0214 | 1.3 | 2.9444 |
| 21 | HMDB0010321 | 3,17-Androstanediol glucuronide | 1.64 | 0.0423 | 1.4 | 11.4964 |
| 22 | HMDB0031162 | 3,3'-Thiobispropanoic acid | 1.6 | 0.0043 | 1.62 | 4.6615 |
| 23 | HMDB0128343 | 3-{3-[3,5-dihydroxy-4-(sulfooxy)benzoyloxy]-4,5-dihydroxybenzoyloxy}-2,4,5-trihydroxybenzoic acid | 1.53 | 0.0173 | 1.54 | 0.5345 |
| 24 | HMDB0000312 | 3a,7a,12b-Trihydroxy-5b-cholanoic acid | 0.62 | 0.0259 | 1.20 | 4.6997 |
| 25 | HMDB0000323 | 3-Amino-2-piperidone | 0.44 | 0.0249 | 1.35 | 0.6120 |
| 26 | HMDB0036442 | 3beta,15alpha-Diacetoxylanosta-8,24-dien-26-oic acid | 0.49 | 0.0008 | 1.84 | 8.6371 |
| 27 | 698 | 3-Methoxy-4-hydroxyphenylethylene glycol | 0.13 | 0.0001 | 1.69 | 3.9545 |
| 28 | HMDB0000459 | 3-Methylcrotonylglycine | 0.36 | 0.0014 | 1.51 | 2.9901 |
| 29 | HMDB0001970 | 3-Methyluric acid | 1.74 | 0.0054 | 1.66 | 3.3776 |
| 30 | 4084 | 3--hydroxy-20-oxopregn-5-en-17--yl sulfate | 2.37 | 0.0489 | 1.02 | 4.0002 |
| 31 | HMDB0061052 | 4-hydroxytriazolam | 1.75 | 0.0000 | 2.11 | 0.7414 |
| 32 | HMDB0030549 | 5,6-Dihydro-4-methoxy-6-[2-(4-methoxyphenyl)ethyl]-2H-pyran-2-one | 2.25 | 0.0429 | 1.38 | 4.4107 |
| 33 | HMDB0004076 | 5-Hydroxykynurenamine | 1.64 | 0.0097 | 1.53 | 2.6483 |
| 34 | HMDB0133722 | 6-{[1-carboxy-8-hydroxy-3,5-dioxo-9-(sulfooxy)-1H,2H,3H,5H-cyclopenta[c]isochromen-7-yl]oxy}-3,4,5-trihydroxyoxane-2-carboxylic acid | 1.52 | 0.0227 | 1.50 | 0.5345 |
| 35 | HMDB0000032 | 7-Dehydrocholesterol | 1.66 | 0.0000 | 2.14 | 9.4473 |
| 36 | LMFA01170053 | 8E-Heptadecenedioic acid | 0.33 | 0.0455 | 1.16 | 5.3887 |
| 37 | HMDB0029765 | 9-Pentadecenoic acid | 1.52 | 0.0017 | 1.75 | 5.4502 |
| 38 | HMDB0014925 | Aciclovir | 1.8 | 0.0399 | 1.33 | 8.0249 |
| 39 | HMDB0035318 | Apiosylglucosyl 4-hydroxybenzoate | 4.06 | 0.0215 | 1.36 | 3.8478 |
| 40 | HMDB0029943 | Arbutin | 1.7 | 0.0061 | 1.79 | 3.1642 |
| 41 | HMDB0028750 | Aspartyl-Cysteine | 1.72 | 0.0000 | 2.19 | 0.5967 |
| 42 | HMDB0001924 | Atenolol | 0.12 | 0.0000 | 1.96 | 6.2511 |
| 43 | HMDB0038232 | Azaspiracid 4 | 1.59 | 0.0063 | 1.44 | 0.5738 |
| 44 | HMDB0014352 | Azithromycin | 0.59 | 0.0004 | 1.78 | 13.8289 |
| 45 | HMDB0000944 | Behenic acid | 1.76 | 0.0078 | 1.60 | 8.4045 |
| 46 | HMDB0006115 | Benzaldehyde | 0.67 | 0.0233 | 1.08 | 2.9367 |
| 47 | HMDB0014452 | Bexarotene | 2.43 | 0.0131 | 1.50 | 3.7422 |
| 48 | 43566 | Biochanin A | 2.38 | 0.0090 | 1.53 | 4.4030 |
| 49 | HMDB0115952 | CDP-DG(16:1(9Z)/18:2(9Z,12Z)) | 1.66 | 0.0261 | 1.4 | 10.6526 |
| 50 | HMDB0001944 | Chlorpheniramine | 0.56 | 0.0335 | 1.38 | 6.2982 |
| 51 | HMDB0010330 | Cholesterol glucuronide | 1.94 | 0.0435 | 1.46 | 9.6614 |
| 52 | HMDB0060644 | cis-Hydroxy Perhexiline | 0.39 | 0.0263 | 1.21 | 0.9929 |
| 53 | 45041 | CMPF | 2.08 | 0.0392 | 1.33 | 3.9850 |
| 54 | HMDB0000601 | Coprocholic acid | 2.1 | 0.0058 | 1.75 | 5.6185 |
| 55 | 43849 | COUMARINIC ACID METHYL ETHER | 1.84 | 0.0218 | 1.39 | 4.6691 |
| 56 | LMSL05000008 | Cuscutic resinoside A | 1.58 | 0.0260 | 1.31 | 3.9164 |
| 57 | HMDB0000656 | Cysteineglutathione disulfide | 2.8 | 0.0000 | 2.05 | 0.6199 |
| 58 | HMDB0028777 | Cysteinyl-Histidine | 1.76 | 0.0020 | 1.72 | 4.6615 |
| 59 | HMDB0014395 | Dapsone | 0.53 | 0.0004 | 1.84 | 0.6817 |
| 60 | HMDB0000122 | D-Glucose | 1.56 | 0.0001 | 1.98 | 0.6120 |
| 61 | HMDB0003701 | Dimethylbenzimidazole | 0.43 | 0.0269 | 1.40 | 13.5241 |
| 62 | HMDB0060897 | Diphenhydramine N-glucuronide | 1.78 | 0.0196 | 1.47 | 9.6299 |
| 63 | HMDB0004158 | D-Urobilinogen | 1.98 | 0.0484 | 1.14 | 3.7117 |
| 64 | HMDB0060898 | Eletriptan N-oxide | 1.8 | 0.0064 | 1.38 | 0.5345 |
| 65 | LMPR0103010023 | farnesyl triphosphate | 1.51 | 0.0058 | 1.64 | 2.8605 |
| 66 | HMDB0015173 | Fenofibrate | 1.96 | 0.0325 | 1.25 | 7.0491 |
| 67 | HMDB0034327 | Glucoconvallasaponin B | 1.6 | 0.0146 | 1.55 | 7.8482 |
| 68 | HMDB0000701 | Hexanoylglycine | 1.64 | 0.0180 | 1.37 | 2.6483 |
| 69 | HMDB0030104 | Humulinic acid A | 3.09 | 0.0079 | 1.40 | 4.4107 |
| 70 | HMDB0013968 | Hydroxyzileuton | 1.72 | 0.0406 | 1.11 | 3.2861 |
| 71 | HMDB0034271 | Hypericin | 1.51 | 0.0031 | 1.50 | 0.6588 |
| 72 | LMPK12111800 | Kaempferol 3-[2''',3''',5'''-triacetyl-alpha-L-arabinofuranosyl-(1->6)-glucoside | 1.83 | 0.0010 | 1.77 | 9.1664 |
| 73 | HMDB0030392 | Kukoamine A | 2.22 | 0.0364 | 1.09 | 4.2963 |
| 74 | 3291 | L-(-)-3-Phenyllactic acid | 2.05 | 0.0120 | 1.41 | 3.4526 |
| 75 | HMDB0011164 | L-beta-aspartyl-L-glutamic acid | 0.36 | 0.0049 | 1.59 | 0.6199 |
| 76 | HMDB0062485 | Lc3Cer | 2.42 | 0.0004 | 2.03 | 10.4321 |
| 77 | HMDB0000148 | L-Glutamic acid | 0.57 | 0.0001 | 1.87 | 0.5890 |
| 78 | HMDB0000222 | L-Palmitoylcarnitine | 1.83 | 0.0092 | 1.54 | 8.8084 |
| 79 | HMDB0000929 | L-Tryptophan | 1.61 | 0.0108 | 1.50 | 2.8529 |
| 80 | HMDB0007852 | LysoPA(0:0/18:2(9Z,12Z)) | 0.54 | 0.0106 | 1.50 | 7.8318 |
| 81 | HMDB0010398 | LysoPC(22:0) | 2.24 | 0.0456 | 1.16 | 9.3285 |
| 82 | HMDB0011487 | LysoPE(0:0/20:4(5Z,8Z,11Z,14Z)) | 1.53 | 0.0008 | 1.70 | 5.7574 |
| 83 | LMFA08020159 | Macamide | 0.62 | 0.0357 | 1.13 | 7.9376 |
| 84 | HMDB0061034 | Metabolite M6 | 2.54 | 0.0003 | 1.82 | 4.5851 |
| 85 | HMDB0000227 | Mevalonic acid | 1.53 | 0.0027 | 1.64 | 3.1947 |
| 86 | HMDB0011545 | MG(0:0/20:3(11Z,14Z,17Z)/0:0) | 1.97 | 0.0012 | 1.84 | 7.3305 |
| 87 | LMGL05010026 | MGDG(16:0/18:2(9Z,12Z)) | 2.25 | 0.0186 | 1.42 | 8.6706 |
| 88 | HMDB0014356 | Midodrine | 0.32 | 0.0403 | 1.26 | 2.8682 |
| 89 | 34535 | N-Acetyl-DL-tryptophan | 2.19 | 0.0006 | 1.70 | 3.4983 |
| 90 | HMDB0000512 | N-Acetyl-L-phenylalanine | 1.81 | 0.0066 | 1.51 | 3.4602 |
| 91 | LMFA08020134 | N-docosahexaenoyl histidine | 2.33 | 0.0107 | 1.41 | 9.5348 |
| 92 | LMPK12111439 | Nevadensin 5-gentiobioside | 1.67 | 0.0066 | 1.50 | 2.8605 |
| 93 | HMDB0012267 | N-Succinyl-L,L-2,6-diaminopimelate | 1.82 | 0.0000 | 2.17 | 0.5193 |
| 94 | LMGP20010032 | OKHdiA-PC | 1.61 | 0.0318 | 1.19 | 5.8571 |
| 95 | HMDB0014343 | Oseltamivir | 0.12 | 0.0000 | 1.92 | 6.9796 |
| 96 | HMDB0240225 | Oxatomide | 10.65 | 0.0034 | 1.56 | 4.2963 |
| 97 | LMGP10030085 | PA(P-20:0/22:4(7Z,10Z,13Z,16Z)) | 1.72 | 0.0013 | 1.68 | 3.4983 |
| 98 | HMDB0000210 | Pantothenic acid | 1.69 | 0.0316 | 1.32 | 2.6483 |
| 99 | HMDB0007880 | PC(14:0/20:2(11Z,14Z)) | 1.98 | 0.0057 | 1.72 | 9.0457 |
| 100 | HMDB0007889 | PC(14:0/22:4(7Z,10Z,13Z,16Z)) | 2.21 | 0.0031 | 1.77 | 11.6248 |
| 101 | HMDB0007936 | PC(15:0/16:1(9Z)) | 0.49 | 0.0136 | 1.44 | 13.8206 |
| 102 | HMDB0007941 | PC(15:0/18:3(6Z,9Z,12Z)) | 0.55 | 0.0100 | 1.43 | 13.8206 |
| 103 | HMDB0007958 | PC(15:0/22:6(4Z,7Z,10Z,13Z,16Z,19Z)) | 1.52 | 0.0100 | 1.55 | 11.2601 |
| 104 | HMDB0007988 | PC(16:0/22:4(7Z,10Z,13Z,16Z)) | 1.97 | 0.0051 | 1.70 | 9.5665 |
| 105 | HMDB0008161 | PC(18:2(9Z,12Z)/P-18:1(11Z)) | 2.38 | 0.0037 | 1.63 | 12.6044 |
| 106 | HMDB0008194 | PC(18:3(6Z,9Z,12Z)/P-18:1(11Z)) | 1.54 | 0.0147 | 1.39 | 13.8125 |
| 107 | HMDB0008292 | PC(20:0/P-18:0) | 1.69 | 0.0013 | 1.69 | 9.4711 |
| 108 | HMDB0008321 | PC(20:1(11Z)/22:6(4Z,7Z,10Z,13Z,16Z,19Z)) | 1.62 | 0.0018 | 1.67 | 10.3707 |
| 109 | HMDB0008392 | PC(20:3(5Z,8Z,11Z)/P-18:1(11Z)) | 2.1 | 0.0048 | 1.65 | 9.2637 |
| 110 | HMDB0008457 | PC(20:4(5Z,8Z,11Z,14Z)/P-18:1(11Z)) | 1.79 | 0.0099 | 1.53 | 11.7544 |
| 111 | HMDB0038568 | PC-M6 | 1.77 | 0.0067 | 1.58 | 4.5086 |
| 112 | HMDB0005779 | PE(O-18:1(1Z)/20:4(5Z,8Z,11Z,14Z)) | 0.55 | 0.0084 | 1.42 | 9.3529 |
| 113 | HMDB0062517 | Pentadecanoylcarnitine | 2.32 | 0.0047 | 1.53 | 9.3770 |
| 114 | HMDB0059586 | Perfluorooctanesulfonic acid | 1.84 | 0.0382 | 1.29 | 6.7479 |
| 115 | LMGP04030092 | PG(P-16:0/22:6(4Z,7Z,10Z,13Z,16Z,19Z)) | 41.81 | 0.0170 | 1.39 | 9.2637 |
| 116 | LMFA03010076 | PGF2alpha isopropyl ester | 1.56 | 0.0010 | 1.71 | 6.9254 |
| 117 | HMDB0000205 | Phenylpyruvic acid | 0.4 | 0.0037 | 1.55 | 11.4507 |
| 118 | LMGP06050012 | PI(22:6(4Z,7Z,10Z,13Z,16Z,19Z)/0:0) | 1.95 | 0.0167 | 1.34 | 6.9100 |
| 119 | LMGP20010013 | PKODiA-PC | 0.38 | 0.0108 | 1.57 | 7.4571 |
| 120 | HMDB0015527 | Prazepam | 1.73 | 0.0003 | 1.99 | 0.5193 |
| 121 | 3557 | Pregnanolone sulfate | 2.69 | 0.0102 | 1.37 | 4.6003 |
| 122 | HMDB0000774 | Pregnenolone sulfate | 3.58 | 0.0086 | 1.37 | 5.0216 |
| 123 | HMDB0012358 | PS(16:0/18:2(9Z,12Z)) | 1.79 | 0.0083 | 1.64 | 8.8002 |
| 124 | HMDB0012394 | PS(18:1(9Z)/20:4(5Z,8Z,11Z,14Z)) | 1.68 | 0.0097 | 1.60 | 13.8125 |
| 125 | LMGP03010477 | PS(19:0/22:2(13Z,16Z)) | 1.6 | 0.0033 | 1.61 | 9.2637 |
| 126 | HMDB0112763 | PS(22:2(13Z,16Z)/15:0) | 1.58 | 0.0494 | 1.07 | 11.2296 |
| 127 | HMDB0112934 | PS(24:1(15Z)/24:1(15Z)) | 1.76 | 0.0055 | 1.68 | 0.5117 |
| 128 | HMDB0061557 | PS(DiMe(11,3)/MonoMe(11,3)) | 0.64 | 0.0072 | 1.53 | 6.0357 |
| 129 | HMDB0000851 | Pyridinoline | 4.82 | 0.0042 | 1.50 | 4.0231 |
| 130 | HMDB0033135 | Pyrimethanil | 2.23 | 0.0032 | 1.57 | 3.0129 |
| 131 | HMDB0013930 | R-95913 | 3.91 | 0.0031 | 1.64 | 3.1413 |
| 132 | HMDB0001930 | Ranitidine | 1.72 | 0.0014 | 1.49 | 0.6356 |
| 133 | HMDB0030263 | Rhazidigenine Nb-oxide | 1.74 | 0.0000 | 1.97 | 0.6356 |
| 134 | HMDB0006239 | S-aminomethyldihydrolipoamide | 1.78 | 0.0034 | 1.56 | 3.1947 |
| 135 | HMDB0037129 | Sanchinoside B1 | 3 | 0.0067 | 1.53 | 10.3553 |
| 136 | HMDB0003219 | Sedoheptulose | 2.08 | 0.0269 | 1.34 | 11.7773 |
| 137 | HMDB0006343 | Selenocystathionine | 1.69 | 0.0064 | 1.62 | 6.0511 |
| 138 | HMDB0013070 | Sinapyl alcohol | 2.88 | 0.0223 | 1.40 | 3.4755 |
| 139 | LMSP03010037 | SM(d16:1/17:0) | 0.63 | 0.0154 | 1.33 | 3.5441 |
| 140 | LMSP03010040 | SM(d16:1/18:1) | 2.2 | 0.0026 | 1.79 | 3.4983 |
| 141 | HMDB0013464 | SM(d18:0/16:1(9Z)) | 0.67 | 0.0289 | 1.21 | 8.4127 |
| 142 | LMSP03010036 | SM(d18:2/15:0) | 1.66 | 0.0026 | 1.59 | 3.5060 |
| 143 | HMDB0000251 | Taurine | 0.66 | 0.0373 | 1.11 | 0.5890 |
| 144 | HMDB0094708 | Tetraethylene glycol | 2.06 | 0.0043 | 1.67 | 3.2633 |
| 145 | HMDB0010357 | Tetrahydroaldosterone-3-glucuronide | 1.67 | 0.0040 | 1.60 | 4.3954 |
| 146 | HMDB0011188 | TG(12:0/12:0/12:0) | 0.64 | 0.0223 | 1.16 | 11.7773 |
| 147 | HMDB0042117 | TG(14:0/15:0/20:5(5Z,8Z,11Z,14Z,17Z)) | 2.35 | 0.0015 | 1.57 | 13.8534 |
| 148 | HMDB0042033 | Thiodiglycol | 0.21 | 0.0090 | 1.39 | 3.1413 |
| 149 | HMDB0000257 | Thiosulfate | 0.3 | 0.0002 | 1.86 | 0.9091 |
| 150 | HMDB0001878 | Thymol | 2.1 | 0.0411 | 1.32 | 6.8493 |
| 151 | 65424 | Trp derivative | 2.07 | 0.0052 | 1.57 | 2.8986 |
| 152 | HMDB0000303 | Tryptamine | 0.29 | 0.0256 | 1.30 | 2.9063 |
| 153 | 44615 | TUBAIC ACID | 2.67 | 0.0285 | 1.34 | 4.9215 |
| 154 | HMDB0004284 | Tyrosol | 0.62 | 0.0013 | 1.67 | 3.1108 |
| 155 | 24059 | D-Glutamylglycine | 0.44 | 0.0288 | 1.31 | 0.6120 |
| 156 | HMDB0015698 | Cinitapride | 0.16 | 0.0000 | 2.17 | 7.0875 |
| 157 | HMDB0029777 | Cymorcin monoglucoside | 0.18 | 0.0000 | 2.17 | 7.0875 |
| 158 | HMDB0030351 | Arborine | 0.12 | 0.0000 | 2.17 | 7.0875 |
| 159 | 201555 | CL (11:0/i-13:0/i-16:0/24:0) | 0.31 | 0.0000 | 2.18 | 7.1028 |
| 160 | HMDB0040939 | Methyl phenyl disulfide | 0.19 | 0.0000 | 2.09 | 7.0875 |
| 161 | HMDB0009928 | PIP(16:0/20:2(11Z,14Z)) | 4.49 | 0.0000 | 2.06 | 4.0428 |
| 162 | HMDB0014652 | Acetyldigitoxin | 2.88 | 0.0000 | 1.97 | 3.8765 |
| 163 | HMDB0003426 | Pantetheine | 2.97 | 0.0000 | 1.93 | 2.7195 |
| 164 | HMDB0010376 | CE(24:0) | 2.16 | 0.0001 | 1.90 | 3.8851 |
| 165 | LMGL02010279 | DG(21:0/22:4(7Z,10Z,13Z,16Z)/0:0)[iso2] | 2.13 | 0.0001 | 1.92 | 3.8765 |
| 166 | HMDB0006492 | 4-Nitrophenyl sulfate | 1.51 | 0.0001 | 2.01 | 6.4200 |
| 167 | LMFA07070024 | (9Z)-3-hydroxydodecenoylcarnitine | 2.13 | 0.0001 | 2.08 | 10.3761 |
| 168 | HMDB0014371 | Guanadrel Sulfate | 1.54 | 0.0001 | 1.93 | 9.6324 |
| 169 | HMDB0041980 | Perchloroethylene | 1.81 | 0.0002 | 1.89 | 0.6079 |
| 170 | HMDB0060730 | 20, 22-Dihydrodigoxigenin | 1.71 | 0.0004 | 2.01 | 10.3999 |
| 171 | HMDB0003929 | 5-Aminoimidazole | 1.87 | 0.0004 | 1.89 | 0.5927 |
| 172 | HMDB0062674 | Bis(2-methylundecan-2-yl) Disulfide | 1.85 | 0.0004 | 1.83 | 9.2140 |
| 173 | 43282 | Octinoxate | 1.94 | 0.0005 | 1.74 | 5.7007 |
| 174 | HMDB0001431 | Pyridoxamine | 1.59 | 0.0005 | 1.83 | 13.8944 |
| 175 | 638 | Chloramphenicol alcohol | 0.5 | 0.0005 | 1.72 | 0.6232 |
| 176 | HMDB0030415 | Quercetin 3-(6''''-feruloylglucosyl)-(1->2)-galactosyl-(1->2)-glucoside | 1.69 | 0.0005 | 1.72 | 4.0955 |
| 177 | 164536 | Ile Pro Pro Val | 1.59 | 0.0006 | 1.80 | 9.2054 |
| 178 | 96523 | 1-Arachidonoyl Glycerol-d5 | 5.28 | 0.0006 | 1.71 | 6.3112 |
| 179 | HMDB0014772 | Sulfacetamide | 0.46 | 0.0009 | 1.69 | 0.8583 |
| 180 | HMDB0013592 | 1,3-Dichloropropene | 0.53 | 0.0010 | 1.77 | 0.7821 |
| 181 | HMDB0014383 | Nevirapine | 2.13 | 0.0013 | 1.66 | 4.9559 |
| 182 | HMDB0028846 | Glycyl-Lysine | 0.49 | 0.0013 | 1.74 | 5.4808 |
| 183 | 58412 | Linoelaidyl carnitine | 0.42 | 0.0014 | 1.74 | 5.4808 |
| 184 | HMDB0011759 | Cer(d18:0/14:0) | 1.59 | 0.0014 | 1.93 | 9.6077 |
| 185 | 137359 | Phe Phe Gln Tyr | 1.58 | 0.0014 | 1.65 | 6.3278 |
| 186 | HMDB0033966 | Di-2-propenyl disulfide, 9CI | 0.67 | 0.0015 | 1.73 | 0.7745 |
| 187 | 132854 | Glu Gln Lys Arg | 7.46 | 0.0015 | 1.75 | 6.3112 |
| 188 | HMDB0042115 | TG(14:0/15:0/18:4(6Z,9Z,12Z,15Z)) | 1.69 | 0.0017 | 1.68 | 8.8380 |
| 189 | 2487 | Stanozolol | 1.66 | 0.0017 | 1.84 | 9.7129 |
| 190 | HMDB0029267 | Kaempferol 3-O-sinapoyl-sophoroside 7-O-glucoside | 2.22 | 0.0017 | 1.59 | 4.0865 |
| 191 | 23685 | Asp Asp | 0.39 | 0.0018 | 1.62 | 0.6232 |
| 192 | LMPR01070141 | 4,4'-Diapophytofluene | 2.21 | 0.0018 | 1.84 | 9.3878 |
| 193 | 70030 | 1,3-Dichloro-2-propanol | 0.62 | 0.0019 | 1.72 | 0.7745 |
| 194 | HMDB0000562 | Creatinine | 0.46 | 0.0019 | 1.59 | 0.6308 |
| 195 | 164596 | Ile Pro Ser Val | 6.42 | 0.0019 | 1.72 | 3.2049 |
| 196 | HMDB0035909 | Glaucarubol 15-O-beta-D-glucopyranoside | 7.77 | 0.0019 | 1.64 | 7.9360 |
| 197 | HMDB0010611 | PG(18:0/22:4(7Z,10Z,13Z,16Z)) | 0.54 | 0.0019 | 1.55 | 13.7742 |
| 198 | 68822 | Pirimicarb | 2.99 | 0.0019 | 1.67 | 2.8631 |
| 199 | 100891 | TG(15:0/16:1(9Z)/18:4(6Z,9Z,12Z,15Z))[iso6] | 2.56 | 0.0020 | 1.65 | 9.8786 |
| 200 | 17161 | Gly His Thr | 0.4 | 0.0021 | 1.59 | 4.1219 |
| 201 | HMDB0009705 | PE(22:6(4Z,7Z,10Z,13Z,16Z,19Z)/22:6(4Z,7Z,10Z,13Z,16Z,19Z)) | 1.51 | 0.0022 | 1.60 | 13.8757 |
| 202 | 106858 | Ala Lys Lys Trp | 19.71 | 0.0022 | 1.65 | 6.3196 |
| 203 | HMDB0014951 | Fentanyl | 3.6 | 0.0023 | 1.63 | 6.3278 |
| 204 | HMDB0004878 | Trihexosylceramide (d18:1/9Z-18:1) | 2.06 | 0.0023 | 1.81 | 0.5117 |
| 205 | 40081 | PC(O-16:0/20:4(5E,8E,11E,14E)) | 1.69 | 0.0024 | 1.63 | 13.8309 |
| 206 | 241736 | Val Gly Pro Val | 4.89 | 0.0024 | 1.67 | 3.1363 |
| 207 | 58401 | Vaccenyl carnitine | 0.62 | 0.0025 | 1.45 | 5.8951 |
| 208 | HMDB0060876 | Thioxanthine monophosphate | 1.54 | 0.0027 | 1.65 | 0.4964 |
| 209 | HMDB0030154 | Austalide G | 2.02 | 0.0028 | 1.72 | 5.7083 |
| 210 | HMDB0014426 | Lidocaine | 3.75 | 0.0031 | 1.66 | 9.3961 |
| 211 | LMGP10050003 | PA(21:4(6Z,9Z,12Z,15Z)/0:0) | 14.14 | 0.0031 | 1.70 | 3.2735 |
| 212 | HMDB0006321 | Docosa-4,7,10,13,16-pentaenoyl carnitine | 0.27 | 0.0031 | 1.58 | 5.5875 |
| 213 | HMDB0032026 | Sinalexin | 2.02 | 0.0031 | 1.58 | 4.6518 |
| 214 | HMDB0041054 | Ustiloxin D | 1.66 | 0.0032 | 1.53 | 4.8580 |
| 215 | HMDB0061835 | Heptaethylene glycol | 8.74 | 0.0032 | 1.64 | 3.0689 |
| 216 | HMDB0061822 | Hexaethylene glycol | 6.23 | 0.0040 | 1.60 | 4.4159 |
| 217 | HMDB0041246 | Secoisotetrandrine | 3.56 | 0.0040 | 1.46 | 8.8720 |
| 218 | 215 | Retinol / Retinol skeleton | 1.84 | 0.0041 | 1.58 | 7.9823 |
| 219 | 89194 | Lotusine | 10.3 | 0.0041 | 1.51 | 6.3278 |
| 220 | HMDB0037158 | 3-[(2-Methyl-3-furanyl)thio]-4-heptanone | 1.66 | 0.0041 | 1.72 | 8.1987 |
| 221 | 59635 | PC(18:3(6Z,9Z,12Z)/22:6(4Z,7Z,10Z,13Z,16Z,19Z)) | 2.03 | 0.0043 | 1.72 | 10.8857 |
| 222 | HMDB0012109 | 5,6-Dihydroxyprostaglandin F1a | 2.07 | 0.0043 | 1.67 | 13.8392 |
| 223 | HMDB0114773 | PA(10:0/13:0) | 1.71 | 0.0044 | 1.62 | 6.4583 |
| 224 | HMDB0014966 | Fosfomycin | 1.62 | 0.0044 | 1.68 | 1.0183 |
| 225 | LMGP06030005 | PI(P-16:0/14:1(9Z)) | 1.54 | 0.0047 | 1.64 | 13.8665 |
| 226 | 39390 | PC(16:0/22:6(3Z,6Z,9Z,12Z,15Z,18))[U] | 2.07 | 0.0050 | 1.73 | 11.2199 |
| 227 | HMDB0007730 | DG(22:5(4Z,7Z,10Z,13Z,16Z)/22:6(4Z,7Z,10Z,13Z,16Z,19Z)/0:0) | 1.94 | 0.0052 | 1.55 | 11.7589 |
| 228 | HMDB15064 | Misoprostol | 84.38 | 0.0054 | 1.47 | 6.3196 |
| 229 | 88253 | Cellulose triacetate | 6.4 | 0.0055 | 1.53 | 6.3196 |
| 230 | 104832 | Ala Glu Ile Pro | 16.56 | 0.0058 | 1.61 | 3.2202 |
| 231 | 88983 | (Z)-3-(1-Formyl-1-propenyl)pentanedioic acid | 1.77 | 0.0059 | 1.68 | 2.6509 |
| 232 | 108536 | Ala Pro Pro Thr | 27.1 | 0.0059 | 1.58 | 3.1516 |
| 233 | HMDB0013943 | Alpha-hydroxyalprazolam | 2.27 | 0.0059 | 1.54 | 2.8174 |
| 234 | HMDB0036062 | Ubiquinone 6 | 2.5 | 0.0060 | 1.56 | 6.2789 |
| 235 | HMDB0015603 | Ximelagatran | 1.59 | 0.0062 | 1.62 | 8.9872 |
| 236 | HMDB0014497 | Methylergonovine | 1.58 | 0.0064 | 1.57 | 5.4198 |
| 237 | 108392 | Ala Pro Gly Pro | 22.08 | 0.0065 | 1.57 | 3.0842 |
| 238 | LMGP04010527 | PG(20:0/22:4(7Z,10Z,13Z,16Z)) | 0.54 | 0.0067 | 1.43 | 13.7424 |
| 239 | HMDB0033336 | Antibiotic X 14889A | 2.36 | 0.0068 | 1.69 | 7.3547 |
| 240 | HMDB0012128 | (R)-Amphetamine | 2.26 | 0.0073 | 1.43 | 0.5770 |
| 241 | HMDB0001368 | 3-Mercaptopyruvic acid | 1.71 | 0.0075 | 1.66 | 2.8174 |
| 242 | HMDB0113009 | PE-NMe(14:1(9Z)/20:5(5Z,8Z,11Z,14Z,17Z)) | 2.19 | 0.0076 | 1.58 | 9.8865 |
| 243 | LMSP01080017 | Plakoside A | 1.6 | 0.0078 | 1.45 | 4.0865 |
| 244 | HMDB0038349 | Goyaglycoside c | 3.69 | 0.0079 | 1.51 | 3.4552 |
| 245 | HMDB0036287 | Campesteryl linoleate | 0.61 | 0.0079 | 1.50 | 7.5761 |
| 246 | HMDB0060597 | Desacetyl-nitazoxanide | 1.59 | 0.0084 | 1.56 | 3.4781 |
| 247 | LMGL05010014 | MGDG(18:3(9Z,12Z,15Z)/18:4(6Z,9Z,12Z,15Z)) | 1.89 | 0.0084 | 1.56 | 12.7919 |
| 248 | HMDB0124854 | {5-[12-(3,4-dihydroxyphenyl)-8,13-dihydroxy-4-oxo-3,11-dioxatricyclo[8.4.0.0,tetradeca-1(10),2(7),8-trien-6-yl]-2-hydroxyphenyl}oxidanesulfonic acid | 2.04 | 0.0087 | 1.71 | 2.8174 |
| 249 | HMDB0030218 | Lansine | 1.89 | 0.0087 | 1.78 | 1.2610 |
| 250 | HMDB0014631 | Altretamine | 1.54 | 0.0090 | 1.63 | 10.8400 |
| 251 | HMDB0034713 | Fumonisin A1 | 1.79 | 0.0091 | 1.47 | 13.1946 |
| 252 | HMDB0012373 | PS(16:1(9Z)/22:6(4Z,7Z,10Z,13Z,16Z,19Z)) | 1.97 | 0.0092 | 1.59 | 10.8781 |
| 253 | LMSP05010060 | GlcCer(d18:1(8E)/18:0(2OH[R])) | 2.25 | 0.0094 | 1.53 | 11.7665 |
| 254 | HMDB0005780 | PE(O-16:1(1Z)/22:6(4Z,7Z,10Z,13Z,16Z,19Z)) | 0.58 | 0.0096 | 1.41 | 10.1207 |
| 255 | HMDB0038390 | Cyclopassifloside III | 1.55 | 0.0098 | 1.60 | 11.1437 |
| 256 | LMSP0501AA59 | GlcCer(d15:2(4E,6E)/20:0) | 2.63 | 0.0098 | 1.55 | 11.9113 |
| 257 | 91883 | Citranaxanthin | 2.14 | 0.0106 | 1.36 | 9.6241 |
| 258 | HMDB0116048 | CDP-DG(18:2(9Z,11Z)/a-15:0) | 1.86 | 0.0107 | 1.46 | 4.0513 |
| 259 | LMPR01070159 | Siphonaxanthin ester/ Siphonaxanthin dodecenoate/ (Siphonein) | 1.96 | 0.0108 | 1.43 | 10.3840 |
| 260 | HMDB0032813 | Isofucosterol 3-O-[6-O-(9-Octadecenoyl)-b-D-glucopyranoside] | 1.7 | 0.0109 | 1.54 | 13.5898 |
| 261 | HMDB0008654 | PC(22:4(7Z,10Z,13Z,16Z)/P-18:1(11Z)) | 1.72 | 0.0111 | 1.60 | 12.8681 |
| 262 | 88933 | 1-(1-Propenylthio)propyl propyl disulfide | 1.63 | 0.0115 | 1.41 | 4.6518 |
| 263 | 15981 | Lys Lys Tyr | 1.59 | 0.0117 | 1.50 | 9.8006 |
| 264 | HMDB0006766 | Estriol-16-Glucuronide | 2.41 | 0.0119 | 1.26 | 13.9130 |
| 265 | HMDB0032380 | (S1)-Methoxy-3-heptanethiol | 2 | 0.012 | 1.38 | 8.8296 |
| 266 | LMST01080071 | 3-O-(Glcb)-6-O-(Glcb)-(25R)-5alpha-spirostan-3beta,6alpha,23S-triol | 1.96 | 0.0122 | 1.57 | 8.8380 |
| 267 | HMDB0007989 | PC(16:0/22:5(4Z,7Z,10Z,13Z,16Z)) | 2.21 | 0.0125 | 1.53 | 11.0598 |
| 268 | HMDB0002362 | 2,4-Diaminobutyric acid | 11.22 | 0.0126 | 1.51 | 2.8784 |
| 269 | HMDB0028720 | Arginyl-Tryptophan | 2.46 | 0.0132 | 1.44 | 11.9711 |
| 270 | HMDB0010666 | PG(18:3(6Z,9Z,12Z)/18:3(6Z,9Z,12Z)) | 1.57 | 0.0137 | 1.65 | 12.2455 |
| 271 | HMDB0072858 | MG(0:0/i-12:0/0:0) | 1.69 | 0.0137 | 1.64 | 9.6809 |
| 272 | 81830 | PA(20:5(5Z,8Z,11Z,14Z,17Z)/19:1(9Z)) | 14.55 | 0.0153 | 1.40 | 6.2613 |
| 273 | HMDB0015215 | Orlistat | 0.59 | 0.0163 | 1.23 | 8.1161 |
| 274 | LMST01120011 | thevetin B | 2.08 | 0.0174 | 1.36 | 10.7332 |
| 275 | HMDB0014555 | Carbachol | 1.59 | 0.0177 | 1.55 | 9.1430 |
| 276 | HMDB0014404 | Sulfanilamide | 1.75 | 0.0180 | 1.25 | 6.0038 |
| 277 | 73392 | (1S,3R)-3-(2,2-Dichloroethenyl)-2,2-dimethylcyclopropanecarboxylate | 1.65 | 0.0180 | 1.27 | 4.6518 |
| 278 | LMFA08020131 | N-arachidonoyl histidine | 2.4 | 0.0185 | 1.64 | 9.6160 |
| 279 | LMGP06010015 | PI(12:0/15:0) | 1.53 | 0.0195 | 1.46 | 11.8275 |
| 280 | LMST01031135 | 18:2-Glc-Campesterol | 1.64 | 0.0198 | 1.52 | 10.7179 |
| 281 | HMDB0033608 | 2-Heptadecylfuran | 0.4 | 0.0198 | 1.27 | 9.8086 |
| 282 | HMDB0014494 | Minoxidil | 1.54 | 0.0198 | 1.45 | 9.6404 |
| 283 | HMDB0010326 | Thyroxine glucuronide | 0.6 | 0.0201 | 1.44 | 6.2701 |
| 284 | HMDB0034994 | Chikusetsusaponin Ia | 1.72 | 0.0203 | 1.43 | 13.0041 |
| 285 | HMDB0112503 | PS(18:4(6Z,9Z,12Z,15Z)/20:5(5Z,8Z,11Z,14Z,17Z)) | 1.78 | 0.0205 | 1.37 | 13.8944 |
| 286 | LMFA07070065 | Glutaconylcarnitine | 0.56 | 0.0214 | 1.12 | 4.1219 |
| 287 | HMDB0033680 | Falcarindione | 1.54 | 0.0232 | 1.18 | 6.3895 |
| 288 | HMDB0029561 | (+)-Calycanthine | 2.05 | 0.0232 | 1.33 | 3.7259 |
| 289 | HMDB0031133 | 1-O-beta-D-Glucopyranosyl-2,3-di-O-(8-hexadecenoyl)glycerol | 2 | 0.0233 | 1.35 | 11.5085 |
| 290 | HMDB0014858 | Clodronate | 1.67 | 0.0234 | 1.45 | 13.8757 |
| 291 | HMDB0007886 | PC(14:0/22:0) | 0.61 | 0.0247 | 1.18 | 9.7209 |
| 292 | HMDB0012217 | Dodecaprenyl diphosphate | 0.61 | 0.0251 | 1.41 | 6.2613 |
| 293 | HMDB0031244 | Isobutylpropylamine | 2.01 | 0.0255 | 1.11 | 9.2054 |
| 294 | 39989 | PC(9:0/10:0)[U] | 0.41 | 0.0256 | 1.28 | 11.8275 |
| 295 | LMFA09000002 | 4Z,7Z,10Z-octadecatrienenitrile | 1.53 | 0.0269 | 1.38 | 4.6136 |
| 296 | LMGP02070003 | PE(P-19:1(12Z)/0:0) | 0.66 | 0.0294 | 1.33 | 6.2701 |
| 297 | 1685 | Propanoic acid, 2-hydroxy-3-[2-(2-propenyloxy)phenoxy]- | 2.78 | 0.0298 | 1.33 | 3.4705 |
| 298 | HMDB0035279 | 8-Methyl-3-hentriacontene | 0.33 | 0.0310 | 1.20 | 8.4752 |
| 299 | HMDB0033154 | 5,6,7,8-Tetrahydroquinoxaline | 0.54 | 0.0325 | 1.30 | 3.6402 |
| 300 | 96370 | 5-ethyl-2-nitro-9h-carbazole | 1.71 | 0.0328 | 1.31 | 3.4705 |
| 301 | HMDB0041080 | 9-Hydroxytridecyl docosanoate | 1.56 | 0.0329 | 1.58 | 9.7369 |
| 302 | HMDB0014667 | Oxaliplatin | 1.95 | 0.0330 | 1.45 | 11.2276 |
| 303 | LMGP06020044 | PI(O-20:0/14:0) | 1.56 | 0.0353 | 1.43 | 10.3604 |
| 304 | HMDB0043220 | TG(15:0/16:1(9Z)/o-18:0) | 1.79 | 0.0357 | 1.38 | 0.5591 |
| 305 | LMFA03120020 | sodium 4R,12S-dihydroxy-9-oxo-5E,7Z,10Z,13Z-prostatetraenoate-cyclo[8,12] | 0.59 | 0.0365 | 1.20 | 3.0842 |
| 306 | 196531 | Asn Pro Pro Pro | 1.57 | 0.0374 | 1.32 | 8.7084 |
| 307 | HMDB0001183 | Octanol | 1.97 | 0.0384 | 1.10 | 1.1772 |
| 308 | HMDB0001920 | Dextromethorphan | 0.35 | 0.0387 | 1.32 | 3.8594 |
| 309 | 67566 | Leptodactylone | 1.66 | 0.0395 | 1.29 | 4.3370 |
| 310 | 60556 | PE(18:3(6Z,9Z,12Z)/P-18:1(11Z)) | 0.64 | 0.0400 | 1.18 | 9.2888 |
| 311 | LMGP20060036 | OHDdiA-PG | 2.2 | 0.0403 | 1.31 | 11.7894 |
| 312 | HMDB0002094 | 3-Fucosyllactose | 2.02 | 0.0403 | 1.23 | 4.1133 |
| 313 | HMDB0042046 | Treosulfan | 1.8 | 0.0403 | 1.29 | 2.8631 |
| 314 | HMDB0041706 | Caffeic acid 3-sulfate | 1.58 | 0.0404 | 1.21 | 0.5193 |
| 315 | 43594 | Valeroyl Salicylate | 2.04 | 0.0414 | 1.33 | 4.3370 |
| 316 | HMDB0035112 | (3beta,17alpha,23R)-17,23-Epoxy-3,29-dihydroxy-27-norlanost-8-ene-15,24-dione | 2.54 | 0.0415 | 1.23 | 5.1847 |
| 317 | HMDB0001063 | 3-Hexaprenyl-4,5-Dihydroxybenzoic acid | 1.72 | 0.0426 | 1.28 | 9.2971 |
| 318 | 1517 | 2-Methyl-3'-hydroxyphenylpropionic acid | 1.99 | 0.0430 | 1.32 | 4.3370 |
| 319 | 4057 | guaifenesin | 0.53 | 0.0435 | 1.18 | 3.0461 |
| 320 | LMGP04060002 | PG(O-18:0/0:0) | 0.59 | 0.0450 | 1.36 | 6.2701 |
| 321 | 263610 | Phenylmalonic acid | 1.65 | 0.0455 | 1.32 | 4.3370 |
| 322 | LMPK04000010 | Erythromycin D | 0.64 | 0.0462 | 1.04 | 8.7084 |
| 323 | 40750 | PE(O-16:0/22:5(4Z,7Z,10Z,13Z,16Z)) | 0.67 | 0.0472 | 1.08 | 9.3299 |
| 324 | LMGP04010587 | PG(20:2(11Z,14Z)/22:4(7Z,10Z,13Z,16Z)) | 0.47 | 0.0475 | 1.14 | 11.7284 |
| 325 | 898 | Oxazepam glucuronide | 1.53 | 0.0482 | 1.17 | 0.6842 |
| 326 | LMGL03011859 | TG(17:2(9Z,12Z)/22:3(10Z,13Z,16Z)/22:3(10Z,13Z,16Z))[iso3] | 0.61 | 0.0491 | 1.25 | 6.2701 |

†: *p* < 0.05 vs. the control.

The blackened font metabolites are the top 20 degree metabolites with metabolite interactions of differential metabolites.

**Table S6. The pathways associated with the differential metabolites**

| Pathsource | Pathway Name | Component Ratio | *P* value | Matched ID |
| --- | --- | --- | --- | --- |
| KEGG | **Retrograde endocannabinoid signaling** | 5/65 | 0.0000 | L-Glutamic acid/PC(16:0/16:0)/PE(O-18:1(1Z)/20:4(5Z,8Z,11Z,14Z))/Sodium/2-Arachidonylglycerol |
| KEGG | Central carbon metabolism in cancer | 4/65 | 0.0022 | D-Glucose/L-Glutamic acid/L-Tryptophan/Fumaric acid |
| KEGG | Amphetamine addiction | 3/65 | 0.0004 | L-Glutamic acid/Sodium/(R)-Amphetamine |
| Reactome | **Amino acid transport across the plasma membrane** | 5/65 | 0.0001 | L-Glutamic acid/L-Tryptophan/Sodium/Taurine/Glycyl-Lysine |
| Reactome | **Amino acid and oligopeptide SLC transporters** | 5/65 | 0.0008 | L-Glutamic acid/L-Tryptophan/Sodium/Taurine/Glycyl-Lysine |
| Reactome | **Transport of inorganic cations/anions and amino acids/oligopeptides** | 5/65 | 0.0013 | L-Glutamic acid/L-Tryptophan/Sodium/Taurine/Glycyl-Lysine |
| Reactome | **Metabolism of polyamines** | 5/65 | 0.0020 | L-Glutamic acid/Sodium/Creatine/Fumaric acid/Creatinine |
| Reactome | Na+/Cl- dependent neurotransmitter transporters | 4/65 | 0.0011 | L-Tryptophan/Sodium/Taurine/Glycyl-Lysine |
| Reactome | Amine compound SLC transporters | 4/65 | 0.0018 | L-Tryptophan/Sodium/Taurine/Glycyl-Lysine |
| Reactome | Acyl chain remodeling of CL | 3/65 | 0.0007 | LysoPC(18:1(9Z))/PC(16:0/16:0)/PE(O-18:1(1Z)/20:4(5Z,8Z,11Z,14Z)) |
| Reactome | Creatine metabolism | 3/65 | 0.0013 | Sodium/Creatine/Creatinine |
| Reactome | **Metabolism of amino acids and derivatives** | 12/65 | 0.0006 | 2-Keto-glutaramic acid/L-Glutamic acid/L-Tryptophan/Phenylpyruvic acid/Sodium/Selenocystathionine/Taurine/C00320/Creatine/Fumaric acid/Creatinine/Glycyl-Lysine |
| Reactome | **Transmembrane transport of small molecules** | 11/65 | 0.0003 | C06810/L-Glutamic acid/L-Tryptophan/Pantothenic acid/PC(16:0/16:0)/PE(O-18:1(1Z)/20:4(5Z,8Z,11Z,14Z))/Sodium/SM(d18:1/18:0)/Taurine/Creatinine/Glycyl-Lysine |
| Reactome | **Signal transduction** | 11/65 | 0.0013 | Arbutin/L-Glutamic acid/L-Palmitoylcarnitine/LysoPC(18:1(9Z))/PC(16:0/16:0)/PE(O-18:1(1Z)/20:4(5Z,8Z,11Z,14Z))/Sodium/SM(d18:1/18:0)/TG(16:0/16:0/18:0)/2-Arachidonylglycerol/Glycyl-Lysine |
| Wikipathways | **Transport of glucose and other sugars, bile salts and organic acids, metal ions and amine compounds** | 6/65 | 0.0015 | Aciclovir/L-Tryptophan/Sodium/Taurine/Creatinine/Glycyl-Lysine |
| Wikipathways | **Transport of inorganic cations-anions and amino acids-oligopeptides** | 5/65 | 0.0009 | L-Glutamic acid/L-Tryptophan/Sodium/Taurine/Glycyl-Lysine |
| Wikipathways | Urea cycle and metabolism of amino groups | 4/65 | 0.0010 | L-Glutamic acid/Creatine/Fumaric acid/Creatinine |
| Wikipathways | Cholesterol biosynthesis | 3/65 | 0.0011 | 7-Dehydrocholesterol/Farnesyl pyrophosphate/Mevalonic acid |
| Wikipathways | **Biochemical pathways part I** | 18/65 | 0.0001 | 7-Dehydrocholesterol/3-Methoxy-4-hydroxyphenylglycol glucuronide/dCMP/D-Glucose/Farnesyl pyrophosphate/L-Glutamic acid/L-Tryptophan/Mevalonic acid/dADP/Pantothenic acid/Phenylpyruvic acid/Taurine/Creatine/Tryptamine/Fumaric acid/Pantetheine/3-Mercaptopyruvic acid/Glycyl-Lysine |

The *p* value of 11 paths is less than 0.05, and the hit component is greater than 5, which has been shown in black.

**Table S7. The gradient elution of mass spectrometry.**

| Time(min) | A% | B% |
| --- | --- | --- |
| 0 | 99 | 1 |
| 1 | 95 | 5 |
| 2 | 70 | 30 |
| 3.5 | 40 | 60 |
| 7.5 | 10 | 90 |
| 9.5 | 0 | 100 |
| 12.5 | 0 | 100 |
| 12.7 | 99 | 1 |
| 16 | 99 | 1 |

A:0.1% formic acid in water

B:0.1% formic acid in acetonitrile

**Table S8. The Parameter of mass spectrometry.**

| Parameter | Positive/Negative ions |
| --- | --- |
| Capillary voltages, Kv | +3, -2 |
| Sampling Cone, V | 40 |
| Source Offset, V | 80 |
| Source Temperature, ℃ | 120 |
| Desolvation Temperature, ℃ | 450 |
| Desolvation Gas Flow, L/h | 800 |
| Cone Gas Flow, L/h | 50 |
| Mass Range, amu | 50-1000 |
| Scan time, s | 0.1 |
| Collision Energy (Low Energy) | 6 |
| Ramp Collision Energy (High Energy) | 20-35 |
| Scan type | MSE centroid |

# Supplement figures


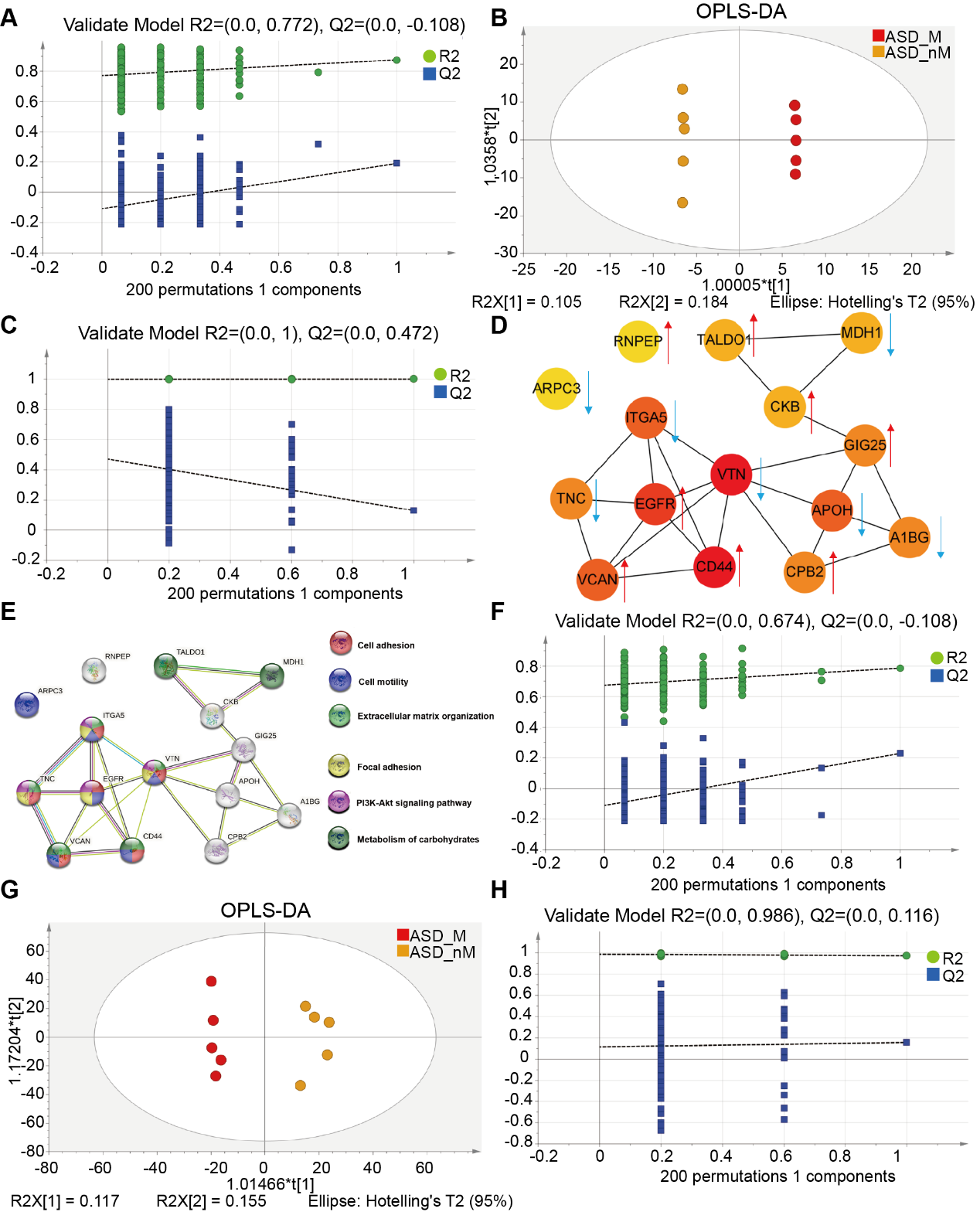


**Figure S1.** **PLS-DA model validation and fifteen hub proteins in the differentially expressed proteins.** (**A**) Validation of the PLS-DA model for total protein analysis by 200 permutation tests. (**B**) OPLS-DA analysis of total proteins in the ASD-M and ASD-nM group. (**C**) Validation of OPLS-DA model by 200 permutation tests. (**D**) PPI network analysis of 15 hub proteins. In hub protein analysis, the darker the color, the higher the score. (**E**) Enrichment analysis of 15 hub proteins by STRING database. Compared with the control group,↑, up-regulated; ↓, down-regulated. (**F**) OPLS-DA analysis of total metabolites in the ASD-M and ASD-nM group. (**G**) Validation of OPLS-DA model by 200 permutation tests. (**H**) Validation of the PLS-DA model for total metabolites analysis by 200 permutation tests.


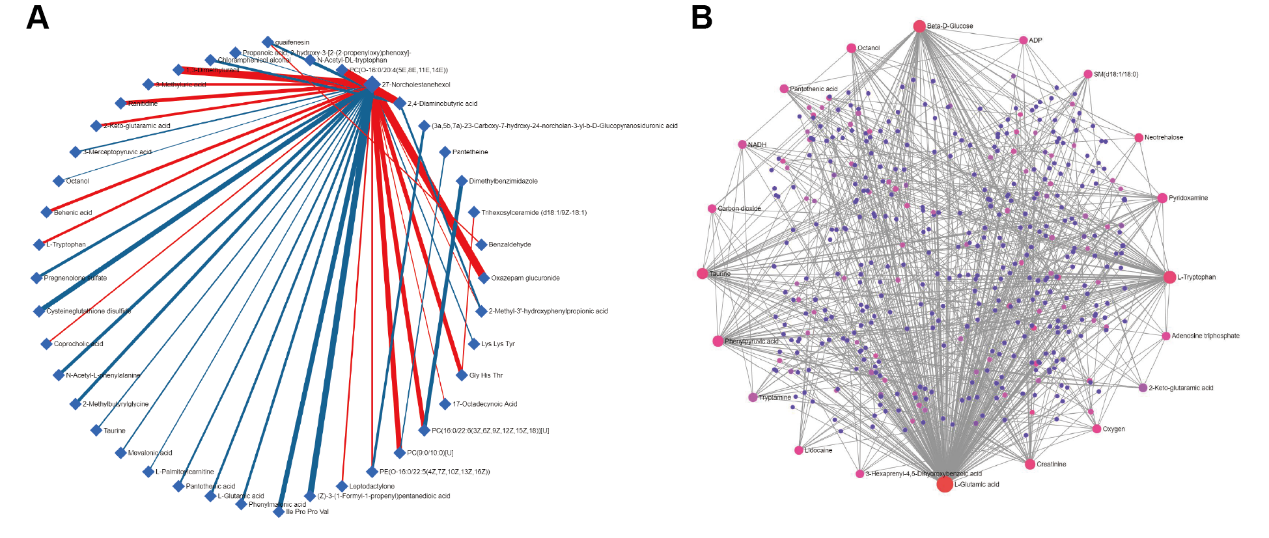


**Figure S2. The metabolite-metabolite interaction network.** (**A**) The correlation analysis of these differential metabolites. The thicker the line, the stronger the correlation. Red represents positive correlation and blue represents negative correlation. (**B**) The top 20 degrees metabolite-metabolite interaction network. Fifteen of them were differential metabolites. Fifteen of them belonged to differential proteins.
